# Identifying the miRNA signature associated with survival time in patients with lung adenocarcinoma using miRNA expression profiles

**DOI:** 10.1038/s41598-017-07739-y

**Published:** 2017-08-08

**Authors:** Srinivasulu Yerukala Sathipati, Shinn-Ying Ho

**Affiliations:** 1 Institute of Bioinformatics and Systems Biology, National Chiao Tung University, Hsinchu, Taiwan; 2 Department of Biological Science and Technology, National Chiao Tung University, Hsinchu, Taiwan

## Abstract

Lung adenocarcinoma is a multifactorial disease. MicroRNA (miRNA) expression profiles are extensively used for discovering potential theranostic biomarkers of lung cancer. This work proposes an optimized support vector regression (SVR) method called SVR-LUAD to simultaneously identify a set of miRNAs referred to the miRNA signature for estimating the survival time of lung adenocarcinoma patients using their miRNA expression profiles. SVR-LUAD uses an inheritable bi-objective combinatorial genetic algorithm to identify a small set of informative miRNAs cooperating with SVR by maximizing estimation accuracy. SVR-LUAD identified 18 out of 332 miRNAs using 10-fold cross-validation and achieved a correlation coefficient of 0.88 ± 0.01 and mean absolute error of 0.56 ± 0.03 year between real and estimated survival time. SVR-LUAD performs well compared to some well-recognized regression methods. The miRNA signature consists of the 18 miRNAs which strongly correlates with lung adenocarcinoma: hsa-let-7f-1, hsa-miR-16-1, hsa-miR-152, hsa-miR-217, hsa-miR-18a, hsa-miR-193b, hsa-miR-3136, hsa-let-7g, hsa-miR-155, hsa-miR-3199-1, hsa-miR-219-2, hsa-miR-1254, hsa-miR-1291, hsa-miR-192, hsa-miR-3653, hsa-miR-3934, hsa-miR-342, and hsa-miR-141. Gene ontology annotation and pathway analysis of the miRNA signature revealed its biological significance in cancer and cellular pathways. This miRNA signature could aid in the development of novel therapeutic approaches to the treatment of lung adenocarcinoma.

## Introduction

Lung cancer has consistently been one of the most lethal cancers. Lung carcinomas are classified into either small-cell lung carcinomas (SCLC) or non-small cell lung carcinomas (NSCLC)^1^. Lung adenocarcinoma is the most common sub-type of NSCLC. Despite advances in cancer therapy, the 5-year survival rate of lung cancer is only 17.4%^2^. Due to the limitation of tumor detection using bronchoscopy and computed tomography techniques^3, 4^, poor early stage detection of lung tumor is a major obstacle to recovery. Therefore, there is a great need of treatment options for NSCLC diagnosis. For accurate detection and potential diagnosis during the NSCLC’s early stage, it is necessary to identify the molecular signature associated with patient survival which may assist in the development of gene target based therapy.

Microarray methods for large-scale analysis of gene expression have helped to systematically identify the molecular biomarkers of cancers^5, 6^. Microarray technology is one of the leading methods for subtyping of cancers on the basis of characteristic expression profiles. Meyerson *et al*. determined the molecular network of lung carcinogenesis by systematically analysing the patient’s protein and mRNA expression profiles^7^. Several researchers focusing on genes and proteins have discovered valuable information such as RB/p16, PTEN, K-ras, FHIT, p53, and MYO18b gene alterations, which are all observed in lung carcinoma^4, 8, 9^. MicroRNAs (miRNAs) are small non-coding RNAs that regulate gene expression and are involved in several biological processes, including human carcinogenesis and embryonic development. Gene and miRNA expression profiles have revealed abundant information on the molecular signatures of various cancer types. MiRNA expression profiles have been used to classify cancers into various subtypes. Many studies reported the relationship between miRNA and cancers, such as colorectal cancer, lung cancer and human cell lymphomas^10, 11^.

Several studies investigated the miRNA expression associated with lung adenocarcinoma. Liu *et al*. distinguished lung adenocarcinoma patients from healthy subjects using miRNA expression profiles and identified seven significantly expressed miRNAs in lung cancer tissue^12^. Yanaihara *et al*. investigated the diagnostic role of miRNAs in lung cancer and reported 43 differently expressed miRNAs in lung cancer tissue when compared with non-cancerous lung tissue^13^. MiRNA expression associated with early stage detection and disease progression was reported in lung cancer^14, 15^. Patnaik *et al*. predicted the recurrence of early stage in 77 NSCLC cases using a support vector machine (SVM) classifier and identified the miRNA expression pattern differentiation in recurrence groups^16^. Yu *et al*. predicted the clinical outcome of 112 NSCLC patients using miRNA expression profiles, and identified five miRNAs that can predict relapse and survival in lung cancer^17^. Raponi *et al*. identified 20 miRNAs that can predict prognosis in 61 squamous cell carcinoma samples using statistical analysis^18^. Besides the well-known survival analysis methods, alternatively, there are linear regression models proposed for survival estimation using censored data^19–21^. Support vector regression methods have been used in medical survival data analysis based on censored data and obtained a significant improvement in accuracy when comparing with standard survival analysis methods^22^. Zhao *et al*. estimated mean survival time using a reinforcement Q-learning method, which is developed based on support vector regression. The reinforcement Q-learning method utilized censored data of patients with advanced metastatic stage IIIB/IV of non-small cell lung cancer. Estimated mean survival time is used as clinical outcome to initiate the second-line therapy in patients with NSCLC^23^.

Current treatment modalities often fail to successfully treat lung cancer though strenuous efforts have been made to find better therapeutics to cure this cancer. Most of these researchers develop microarray methods to identify tumors and cancer stages. MiRNAs exceptionally influence developmental and oncogenic pathways by regulating gene expression^11, 24^. Defects in the miRNA biogenesis mechanism cause oncogenesis in lung cancer. Kumar *et al*. reported that conditional deletion of Dicer 1 associated with the lung tumor development in a mouse model^25^. Reduced dicer expression is involved in the development of lung tumors and shows a significant prognostic impact on survival of lung cancer patients^26^. A collection of evidences shows that miRNAs are differently expressed in non-small cell lung cancers and act as tumor suppressor and oncogenes^27^. For example, hsa-let-7 family often acts as a tumor suppressor. Hsa-let-7 family was found to be frequently deleted in chromosomal regions of lung cancer cell lines^28^ and inhibition of let-7 expression leads to increased cell division in A549 lung cancer cell lines^29^. Additionally, let-7 family negatively regulates oncogenes such as MYC and RAS^25, 30^. MiRNAs such as miR-31 function as oncogenic miRNAs in lung cancer by suppressing the tumor suppressor genes PP2A regulatory subunit B alpha isoform and large tumor suppressor-2^31^. MiR-17-92 cluster promotes cell proliferation in non-small cell lung cancer^32^. Furthermore, miRNAs such as miR-1244 are down-regulated in A549 cells and involved in the progress of cisplatin resistance in NSCLC^33^. MiR-630 controls the p-53 regulated pro-apoptotic pathway and is involved in chemo resistant determination in lung cancer cells^33^. These studies imply a significant role of miRNAs in the development and progression of lung cancers.

MiRNA expression profiles are helpful to identify survival-related variants of lung adenocarcinoma. The miRNA signature associated with patient survival is also helpful for the development and evaluation of gene target based therapy. However, few studies develop machine learning approaches to identify the miRNA signature of patient survival^34^. Accordingly, the aim of this work is to identify the miRNA signature that can predict patients’ survival time in lung adenocarcinoma. This work proposes a support vector regression (SVR) based predictor, SVR-LUAD, to identify the miRNA signature associated with the survival time of patients with lung adenocarcinoma. Estimating survival time is very important, especially in cancer studies, to evaluate the personalized treatment effects in individuals with cancer. Identification of miRNA signature associated with survival time will help to further understand the miRNA mechanism in lung cancer as well as develop the therapeutics.

The SVR-LUAD method uses an optimal feature selection method, an inheritable bi-objective combinatorial genetic algorithm (IBCGA)^35^ to identify a small set of informative miRNA cooperating with SVR by maximizing estimation accuracy of survival time. There were 102 lung adenocarcinoma patients’ miRNA expression profiles along with survival information extracted from the cancer genome atlas (TCGA) database. SVR-LUAD identified a set of 18 miRNAs from the expression profiles of 332 miRNAs. The 18-miRNA signature is highly associated with lung adenocarcinoma survival. SVR-LUAD achieved a correlation coefficient of 0.88 ± 0.01 and mean absolute error of 0.56 ± 0.03 year (mean and standard deviation) between the real and estimated survival time using 10-fold cross-validation (10-CV). We validated the SVR-LUAD method using an independent test cohort of 51 lung adenocarcinoma patients obtained from the TCGA database. Additionally, we analysed the 18-miRNA signature using gene ontology (GO) and Kyoto Encyclopedia of Genes and Genomes (KEGG) pathway. These findings may be helpful towards understanding the individual miRNA role in lung adenocarcinoma survival.

## Results and Discussion

SVR-LUAD simultaneously identified the miRNA signature and estimated the survival time of lung adenocarcinoma patients using miRNA expression profiles. Based on the accurate estimation of survival time, we can further understand individual miRNAs of the signature. There were 102 and 51 patients along with expression profiles of 332 miRNAs for training and testing the prediction model.

### Identifying the miRNA signature with survival time estimation

SVR-LUAD used an optimal feature selection algorithm IBCGA to identify a set of 18 informative miRNAs (referred to a miRNA signature) associated with the estimation of lung adenocarcinoma survival time. Since the optimal feature selection method IBCGA is a non-deterministic method, we employed 30 independent runs to select one robust feature set. The 30 runs and their corresponding appearance scores are shown in Supplementary Table S1. We compare the SVR-LUAD method with penalized regression methods, such as LASSO, Ridge regression, Elastic net, and Multiple linear regression. The Elastic net is a combination of both methods LASSO and Ridge regression. The comparison results of SVR-LUAD, Elastic net and Multiple linear regression methods are shown in Table 1.

**Table 1:** Prediction performance comparison of SVR-LUAD.

Method	MiRNAs selected	Correlation coefficient	Mean absolute error
Multiple linear regression	5	0.53	0.99
SVR-LUAD-5	5	0.66	0.94
Elastic net	8	0.55	1.02
SVR-LUAD-8	8	0.72	0.81
SVR-LUAD	18	0.90	0.52
SVR-LUAD-mean	24.7	0.88	0.56

**Figure 1:**
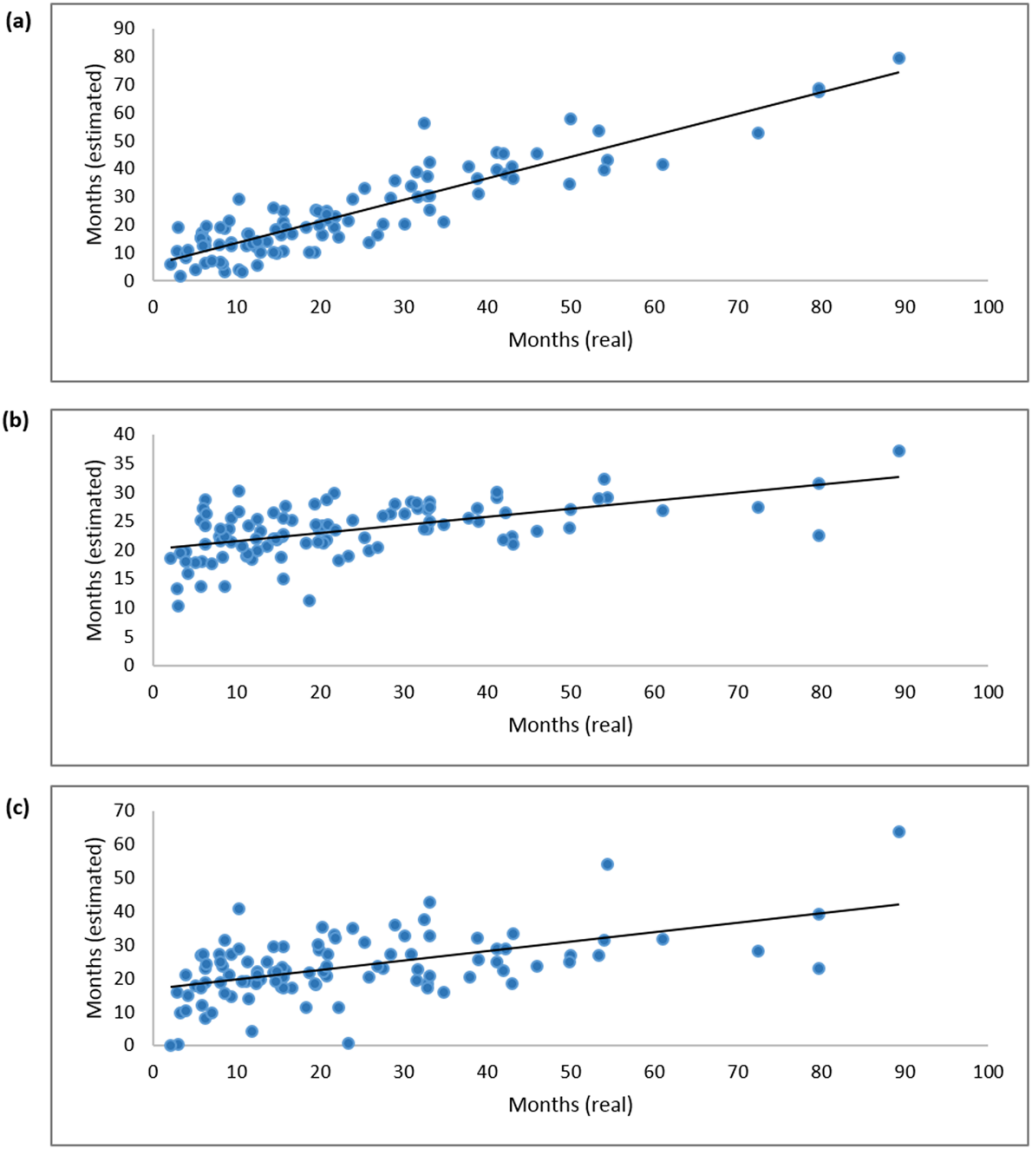
(**a)** Prediction performance of SVR-LUAD with a correlation coefficient of 0.90. **(b)** Prediction performance of Elastic net with a correlation coefficient of 0.55. **(c)** Prediction performance of Multiple linear regression with a correlation coefficient of 0.53. X-axis refers to real survival time and Y-axis refers to estimated survival time.

The highest appearance score of SVR-LUAD was 0.53 (=16.0/30) with 18 miRNAs indicating that each miRNA of the signature may be selected with the probability of 0.53 in a run of SVR-LUAD on average (Fig. S1). The estimation accuracy of SVR-LUAD was the correlation coefficient of 0.90 and mean absolute error of 0.52 year using 10-CV. SVR-LUAD-mean achieved a correlation coefficient of 0.88 ± 0.01 and mean absolute error of 0.56 ± 0.03 year on average. The LASSO method achieved a correlation coefficient and mean absolute error of 0.48 and 1.083 year, and Ridge regression achieved a correlation coefficient and mean absolute error of 0.51 and 1.086 year, respectively. The Elastic net method with 8 miRNAs achieved a correlation coefficient and mean absolute error of 0.55 and 1.02 year, and Multiple linear regression with 5 miRNAs obtained a correlation coefficient and mean absolute error of 0.53 and 0.99 year, respectively. To fairly compare SVR-LUAD with these methods, the feature numbers of SVR-LUAD were restricted to 5 and 8. SVR-LUAD-5 with 5 miRNAs obtained a correlation coefficient and mean absolute error of 0.66 and 0.94 year, respectively. SVR-LUAD-8 with 8 miRNAs obtained a correlation coefficient and mean absolute error of 0.72 and 0.81 year, respectively. The correlation plots of the real and estimated survival time for SVR-LUAD, Elastic net, and Multiple linear regression are shown in Fig. 1. The correlation plots for LASSO and Ridge regression are shown in Supplementary Fig. S2. SVR-LUAD is better than these compared methods.

### Evaluation on an independent test cohort

**Figure 2:**
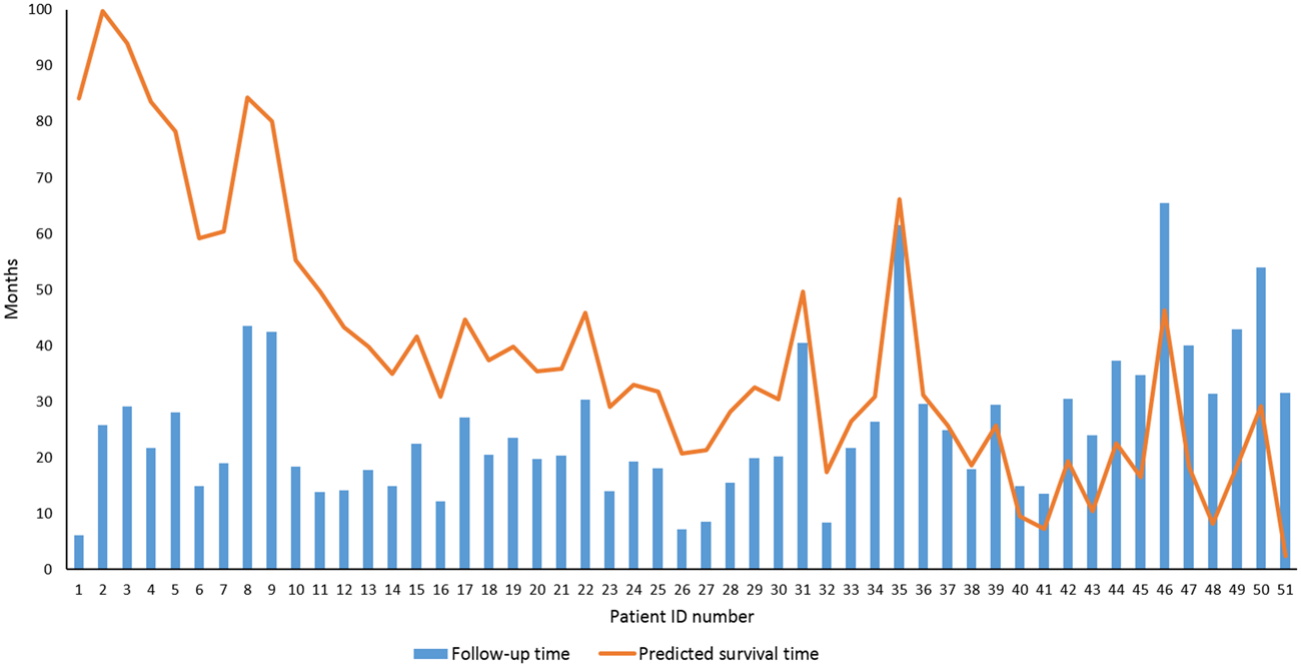
The validation of SVR-LUAD on an independent cohort of 51 patients with lung adenocarcinoma. Predicted survival time is larger than the follow-up time for the first 38 patients (1–38).

The 51 patients with follow-up time are alive and have tumors after therapy. The prediction result of SVR-LUAD for individual patients is shown in Fig. 2. The mean and standard deviation of the follow-up time are 25.27 ± 12.95 months. SVR-LUAD predicted and obtained the survival time of 38.92 ± 23.68 months. There were 38 of the 51 (75%) patients whose predicted survival time is larger than his/her follow-up time. The predicted survival time and follow-up time of the remaining 13 patients were 18.01 ± 11.52 and 34.57 ± 14.27 months on average, respectively. Comparing to the prediction error of 0.52 years (Table 1) and the mean difference, 16.56 (=34.57–18.01) months, of follow-up time and predicted survival time, it reveals that the pharmaceutical therapy may be promising.

### Contribution of individual miRNAs

SVR-LUAD with the 18-miRNA signature can achieve high estimation accuracy, but it doesn’t mean that the 18 miRNAs are the only informative miRNAs. For example, some miRNAs (e.g., hsa-let-7a-2, hsa-miR-192, hsa-miR-20b, hsa-miR-24-1, hsa-miR-25, hsa-miR-3187, hsa-miR-34b, hsa-miR-3617 and hsa-miR-1254, hsa-miR-1291, hsa-miR-194-2, hsa-miR-212, hsa-miR-3920) obtained from SVR-LUAD-8 and SVR-LUAD-5 don’t belong to the 18-miRNA signature. An increase of the patient cohort may be helpful to improve the prediction accuracy and identify the really informative miRNAs associated with survival time of patients with lung adenocarcinoma. We evaluated the contribution of individual miRNAs towards the estimation of survival time using two analysis methods, main effect difference and individual miRNA effect. The 10 top-ranked miRNAs according to the contribution of survival time estimation were further analyzed.

### Main effect difference

We measure the individual effect of each miRNA in the 18-miRNA signature using an orthogonal array experimental design^36^. The larger the value of the main effect difference^37^, the larger the contribution of this miRNA toward to the survival time prediction using the signature is. The 10 top-ranked miRNAs are hsa-let-7f-1, hsa-miR-16-1, hsa-miR-152, hsa-miR-217, hsa-miR-18a, hsa-miR-193b, hsa-miR-3136, hsa-let-7g, hsa-miR-155 and hsa-miR-3199-1. Hereafter these will be referred to as top-10. All the 18 miRNAs and their corresponding main effect difference are shown in Table 2.

**Table 2:** Contribution of individual miRNAs using MED and individual miRNA effect.

Rank	MiRNA	MED	MiRNA effect	MAE (month)
		value	Correlation coefficient	
1	**hsa-let-7f-1**	1.790	0.25	12.95
2	**hsa-miR-16-1**	1.575	0.60	9.43
3	**hsa-miR-152**	0.966	0.07	13.24
4	**hsa-miR-217**	0.955	0.35	12.05
5	**hsa-miR-18a**	0.952	0.42	11.57
6	**hsa-miR-193b**	0.921	0.61	8.74
7	**hsa-miR-3136**	0.779	0.29	12.08
8	**hsa-let-7g**	0.775	0.57	9.63
9	**hsa-miR-155**	0.622	0.35	12.13
10	**hsa-miR-3199-1**	0.446	0.27	12.69
11	**hsa-miR-219-2**	0.407	0.29	11.88
12	**hsa-miR-1254**	0.398	0.49	10.75
13	**hsa-miR-1291**	0.396	0.54	11.05
14	**hsa-miR-192**	0.324	0.30	12.71
15	**hsa-miR-3653**	0.274	0.47	11.30
16	**hsa-miR-3934**	0.234	0.46	10.73
17	**hsa-miR-342**	0.216	0.38	11.05
18	**hsa-miR-141**	0.033	0.43	10.08

### Individual miRNA effect

We assess the ability of an individual miRNA in estimating survival time of cancer patients using this miRNA only. We take one miRNA from the 18-miRNA signature and obtain the estimation performance of this miRNA in terms of correlation coefficient and mean absolute error, shown in Table 2. The results show that the three miRNAs, hsa-miR-193b, hsa-miR-16-1 and hsa-let-7g, have the highest correlation coefficients 0.61, 0.60 and 0.57 and mean absolute errors 8.74, 9.43 and 9.63 months, respectively. The ability of a miRNA in estimating survival time is slightly different between the roles in a signature and a single miRNA. Correlation plots for each miRNA are shown in Supplementary Fig. S3. The top-10 miRNAs are discussed below.

Among top-10 miRNAs, most of the miRNAs are involved in several major cancer types including lung cancer, bladder cancer, breast cancer, hepatocellular carcinoma, glioblastoma, ovarian and gastric cancers. We summarize the top-10 miRNAs and their involvement in various cancer types in Table 3.

**Table 3:** The top-10 miRNAs involved in various cancers.

MiRNA	Cancer	Regulation	Reference
hsa-let-7f-1	Lung cancer	down	25, 30, 38
	Breast cancer	down	102
	Colon cancer	down	103
	Hepatocellular carcinoma	down	104
	Neuroblastoma	down	105
	Pancreatic ductal adenocarcinoma	down	106
hsa-miR-16-1	Lung cancer	down	42, 43
	Prostate cancer	down	107
	Neuroblastoma	up	108
	Chronic lymphocytic leukemia	down	109
hsa-miR-152	Lung cancer	down	44, 48, 49
	Breast cancer	down	49
	Colorectal cancer	down	110
	Glioblastoma	down	111
	Ovarian cancer	down	112
hsa-miR-217	Lung cancer	down	113
	Breast cancer	up	114
	Gastric cancer	down	115
	Hepatocellular carcinoma	up	116
	Pancreatic ductal adenocarcinoma	down	117
hsa-miR-18a	Lung cancer	up	32, 54
	Colorectal cancer	up	118
	Colon cancer	up	119
	Gastric cancer	up	120
hsa-miR-193b	Lung cancer	down	55–57
	Breast cancer	down	121
	Gastric cancer	down	122
	Hepatocellular carcinoma	down	123
	Ovarian cancer	down	124
hsa-miR-3136	Acute myeloid leukemia	—	67
	Esophageal adenocarcinoma	—	125
	Breast cancer	—	68
hsa-let-7g	Lung cancer	down	58–60
	Breast cancer	down	102
	Colon cancer	down	103
	Esophageal squamous cell carcinoma	down	126
	Hepatocellular carcinoma	down	104
hsa-miR-155	Lung cancer	up	50, 63, 64
	Acute myeloid leukemia	up	37
	Bladder cancer	up	127
	Breast cancer	up	128
	Nasopharyngeal carcinoma	up	129
hsa-miR-3199–1	Prostate cancer	up	69
	Breast cancer	—	130

### Roles of the identified miRNAs

We analysed individual roles of the top-10 miRNAs in the 18-miRNA signature. Among the top-10 miRNAs, eight miRNAs are involved not only in lung cancer but also in other major cancer types. The two miRNAs hsa-miR-3136 and hsa-miR-3199-1 are involved in tumorigenesis but they are not reported as being involved in lung cancer.

(1) Hsa-let-7f-1: According to the MED analysis, hsa-let-7f-1 with rank 1 is the most effective in estimating survival time of lung adenocarcinoma patients. SVR-LUAD achieved a correlation coefficient of 0.25 and MAE of 12.95 months revealing that hsa-let-7f-1 is also very informative associated with the survival time. The hsa-let-7 family functionally inhibits oncogenes, such as c-Myc^25^, the Ras family^30^, and HMGA2^38^. Hsa-let-7f-1 often acts as a tumor suppressor and is down-regulated in NSCLC due to its reduced expression^39^. The genes of the hsa-let-7 family are frequently located in the genomic regions of human cancers^28^. Takamizawa *et al*. investigated 143 lung cancer cases and reported that the reduced expression of the hsa-let-7 family is significantly associated with poor prognoses of lung cancer patients^40^. Real-time RT-PCR analysis on hsa-let-7a revealed that the expression of hsa-let-7 in lung cancer patients is significantly different (p = 0.000398) from the non-cancerous lung tissue; and altered expression of hsa-let-7a is associated with poor survival in lung adenocarcinoma patients^13^. Significance analysis on microarray studies reported that hsa-let-7f-1 is differently expressed in plasma of NSCLC patients (mean 0.53 fold change) when compared with healthy controls^41^. Altogether, these findings suggest that expression alteration of hsa-let-7f-1 has a prognostic impact on lung adenocarcinoma patients.

(2) Hsa-miR-16-1: Experimental investigation on NSCLC reported that hsa-miR-16-1 is mostly down-regulated in lung adenocarcinoma cell lines, Calu-1, H2009, H1299, H358 and A549, and this miRNA induced cell cycle arrest^42^. Normalized PCR experimental results of 70 NSCLC patients showed that hsa-miR-16-1 was down-regulated in tumor tissue (p < 0.001) when compared with normal tissue^43^. A clinical level study of 77 surgically-treated NSCLC patients found that hsa-miR-16-1 over expressed (2.650 fold change) in a recurrence group when compared with no-recurrence cases^16^.

(3) Hsa-miR-152: Chen *et al*. reported that hsa-miR-152 was up-regulated in NSCLC patients serum compared with control subjects’ serum^44^. Su *et al*. reported that hsa-miR-152 is down-regulated in NSCLC cells and acts as a tumor suppressor^45^. Hsa-miR-152 induces cell proliferation, migration, invasion and colony formation by targeting ADAM metallopeptidase domain 17 in NSCLC tissue^45^. Down-regulated expression of hsa-miR-152 targets the neuropilin-1 and regulates cancer metastasis in NSCLC A549 cell lines^46^. Hsa-miR-152 expression is significantly down-regulated in NSCLC patients when comparing with the healthy controls^47^. This miRNA was also majorly involved in cancer types such as epithelial ovarian^48^ and breast cancers^49^.

(4) Hsa-miR-217: Real-time PCR study on 100 patients revealed that over expression of hsa-miR-217 inhibited the cell proliferation, migration and promoted the apoptosis in lung cancer cells^50^. Hsa-miR-217 expression was significantly lower in lung cancer tissue when compared to the normal tissue^50^. Hsa-miR-217 has an emerging role in pancreatic ductal adenocarcinoma^51^, and its expression was up-regulated in B-cell lymphocytic leukemia^52^. Hsa-miR-217 targets phosphatase and tensin homologue, resulting in the activation of Akt kinase in diabetic nephropathy^53^.

(5) Hsa-miR-18a: Hayashita *et al*. reported that hsa-miR-18 is overexpressed in the amplified chromosomal region of lung cancer^32^. Its expression was significantly higher (20.25 fold change) in high-grade neuroendocrine pulmonary tumors of the lungs^54^.

(6) Hsa-miR-193b: Hsa-miR-193b was up-regulated and differentially expressed (1.50 fold change) in short survival versus long survival NSCLC patients^55^. A microarray study on 38 NSCLC patients reported that hsa-miR-193b is up-regulated in lung cancer tissue with a 6.8 fold change when compared with the normal tissue^56^. Quantitative RT-PCR analysis reported that hsa-miR-193b expression was lower in the NSCLC cell line A549 when compared with normal tissue, and that it modulates cell migration, invasion, and proliferation in NSCLC cells^57^.

(7) Hsa-let-7g: The hsa-let-7 family plays a key role in lung cancer. Hsa-let-7g inhibits tumor cell proliferation and promotes tumor cell death as observed in murine K-Ras expressing lung adenocarcinoma cells (LKR13)^58^. This miRNA can actively suppress the tumor formation in K-Ras mutant NSCLC cell lines^58^. Single nucleotide poly morphisms potentially modify the hsa-let-7g binding and target gene regulation, which eventually increases the risk of NSCLC cells^59^. Park *et al*. reported that hsa-let-7g is aberrantly expressed in NSCLC cells. Further-more, hsa-let-7g targets the genes HMGA2 and K-Ras resulting in the inhibition of A549 lung cancer cell migration^60^. This miRNA’s expression inhibits the nuclear-factor kappa B1 and plays a significant role in enhancing the ability of radiotherapy in lung cancer^61^. Joeng *et al*. investigated the role of hsa-let-7g in radio-sensitivity in lung cancer, and reported that over expression of this miRNA enhances radio-sensitivity in radio-resistant H1299 lung cancer cells, and also increases the response to ionizing radiation^62^.

(8) Hsa-miR-155: Yanaihara *et al*. investigated miRNA expression associated with the lung cancer patient survival and found that higher expression of hsa-miR-155 is associated with poor survival in lung adenocarcinoma patients. Its expression was significantly different in lung cancer tissue (p = 1.00e-07) when compared with non-lung cancer tissue^13^. A real time PCR study of 74 lung cancer patients reported that hsa-miR-155 expression was significantly higher in lung cancer tissue than control tissue (p < 0.001) and this miRNAs expression was used to discriminate the lung cancer cells from controls^63^. Volinia *et al*. reported higher expression of hsa-miR-155 in lung, breast and colon cancers^64^. Hsa-miR-155 was reported as a significant cancer regulator and it is under-expressed in lung cancer cells and other cancers^50^.

(9) Hsa-miR-3136 and hsa-miR-3199-1: He *et al*. reported that single nucleotide polymorphism occurs between hsa-miR-3136 and 3′ UTR of lung cancer-related inflammatory gene KSR1 with a mean allele frequency of 0.293^65^. Hsa-miR-3136 is involved in Klinefelter syndrome^66^ and childhood acute lymphoblastic leukemia^67^, and its over expression is observed in breast cancer^68^. Hsa-miR-3199-1 is involved in castration resistant prostate cancer^69^. According to the individual miRNA effects, the two miRNAs, hsa-miR-3136 and hsa-miR-3199-1, have correlation coefficients of 0.29 and 0.27, respectively, that means their contribution towards survival prediction is relatively high in lung adenocarcinoma.

Additionally, we investigated the miRNA expression differentiation between lung cancer tissue and normal tissue using the TCGA database, in which 473 cancer cases and 45 controls were used^70^. Hsa-miR-3136 expression is up-regulated in lung cancer when compared to the normal tissue with a fold change of 0.093 and a p-value of 0.009. Hsa-miR-3199-1 expression is down-regulated in lung cancer with a fold change of −0.12 and a p-value of 0.002 between cancer and normal tissue^70^. Hence, this work found that the two miRNAs have significant association with lung adenocarcinoma survival and should be further investigated for their roles in lung adenocarcinoma.

We summarize the top-10 miRNAs and their functions as either oncogenic or tumor-suppressor in Table 4. We constructed a miRNA-target interaction network using the CyTargetLinker application, supported by the Cytoscape^71^ to explore the regulatory interactions derived from interaction databases. The top-10 miRNAs are annotated with miRBase accession numbers, and identified 7654 predicted miRNA-target interactions. In the predicted network, only the miRNA-target interaction network in MicroCosm and TargetScan are visualized for the top-10 miRNAs, shown in Supplementary Fig. S4. The experimentally validated target genes of the top-10 miRNAs were reported in supplementary Table S2 from DIANA-TarBase^72^ and existing studies. We describe the top-10 miRNAs with some selected target genes using the validation methods including immunohistochemistry, western blot analysis, qPCR and immunoprecipitation below.

**Table 4:** Role of the top-10 miRNAs in lung cancer.

Rank	MiRNAs	Oncogenic/Tumor suppressor	Reference
1	hsa-let-7f-1	Tumor-suppressor	25, 30, 38, 39
2	hsa-miR-16-1	Tumor-suppressor	42, 131
3	hsa-miR-152	Tumor-suppressor	45, 46
4	hsa-miR-217	Tumor-suppressor	132
5	hsa-miR-18a	Oncogenic	133
6	hsa-miR-193b	Tumor-suppressor	57
7	hsa-miR-3136	—	—
8	hsa-let-7g	Tumor-suppressor	58–60
9	hsa-miR-155	Oncogenic	13
10	hsa-miR-3199-1	—	—

There were five miRNAs with validation using immunohistochemistry, western blot analysis and qPCR which are hsa-let-7f-1, hsa-miR-217, hsa-miR-18a, hsa-let-7g and hsa-miR-155. The hsa-let-7 family targets PRDM1 and regulates its function in a diffuse large B-cell lymphoma^73^. Hsa-miR-217 targets E2F3 which is positively associated with the hepatocellular carcinoma metastasis^74^. Hsa-miR-18a alters the PTEN protein expression in neural progenitor cells^75^. Hsa-let-7g is one of the hypoxia-responsive miRNAs targeting argonaute 1, which controls the miRNA induced silencing complex^76^. Hsa-miR-155 down-regulates the JMJD1A and BACH1 expression in nasopharyngeal cell lines^77^.

There were eight miRNAs with validation on their functional targets using western blot analysis and qPCR. Besides the five miRNAs mentioned above, the other three miRNAs were hsa-miR-16-1, hsa-miR-152 and hsa-miR-193b. For instance, hsa-miR-16-1 regulates the expression of CyclinD1 (CCDN1), which is an important regulator of cell-cycle progression^78^. Inhibition of miR-152 regulates DNA methylation via targeting DNA methyl transferase 1 in nickel sulfide transformed human bronchial epithelial cells^79^. Hsa-miR-193b directly targets the oncogenes CCD1 and ETS1 and regulates the invasion and migration in hepatoma cells^80^. The studies with immunoprecipitation experiments reported that hsa-miR-3136 targets SOCS5 with down-regulation in lymphoblastoid cell lines^81^ and hsa-miR-3199-1 targets CDK16 in 293 C cell lines^82^.

Aside from the top-10 miRNAs, other identified miRNAs such as hsa-miR-219-2, are down-regulated and differently expressed (p = 5.56e-05) in lung cancer cells when compared with non-lung cancer cells^13^. Foss *et al*. investigated differently expressed serum-based miRNAs in NSCLC, and found that hsa-miR-1254 expression was significantly higher in early stage NSCLC cells; they then used this miRNA to distinguish early stage NSCLC samples from controls^83^. Hsa-miR-192 is down-regulated in lung cancer cells and its expression was significantly higher (p = 0.000119) in lung cancer tissue when compared with non-lung cancer tissues^13^. Overexpression of hsa-miR-192 suppresses cell proliferation in NSCLC cells and it is usually down-regulated in NSCLC cells when compared with adjacent non-cancer cells^84^. It has been reported that hsa-miR-141 is differently expressed in primary lung tumors^85^.

### Biological significance of the top-ranked miRNAs

We investigated whether the selected miRNAs have biological significance in cellular pathways and molecular functions using two analysis procedures, the KEGG pathway analysis and gene ontology terms using the Diana tool. First, we employed the KEGG pathway analysis for the top-10 miRNAs. In this analysis, the top-10 miRNAs are functionally enriched in a total of 60 cancer/non-cancer KEGG pathways, shown in Supplementary Table S3.

**Figure 3:**
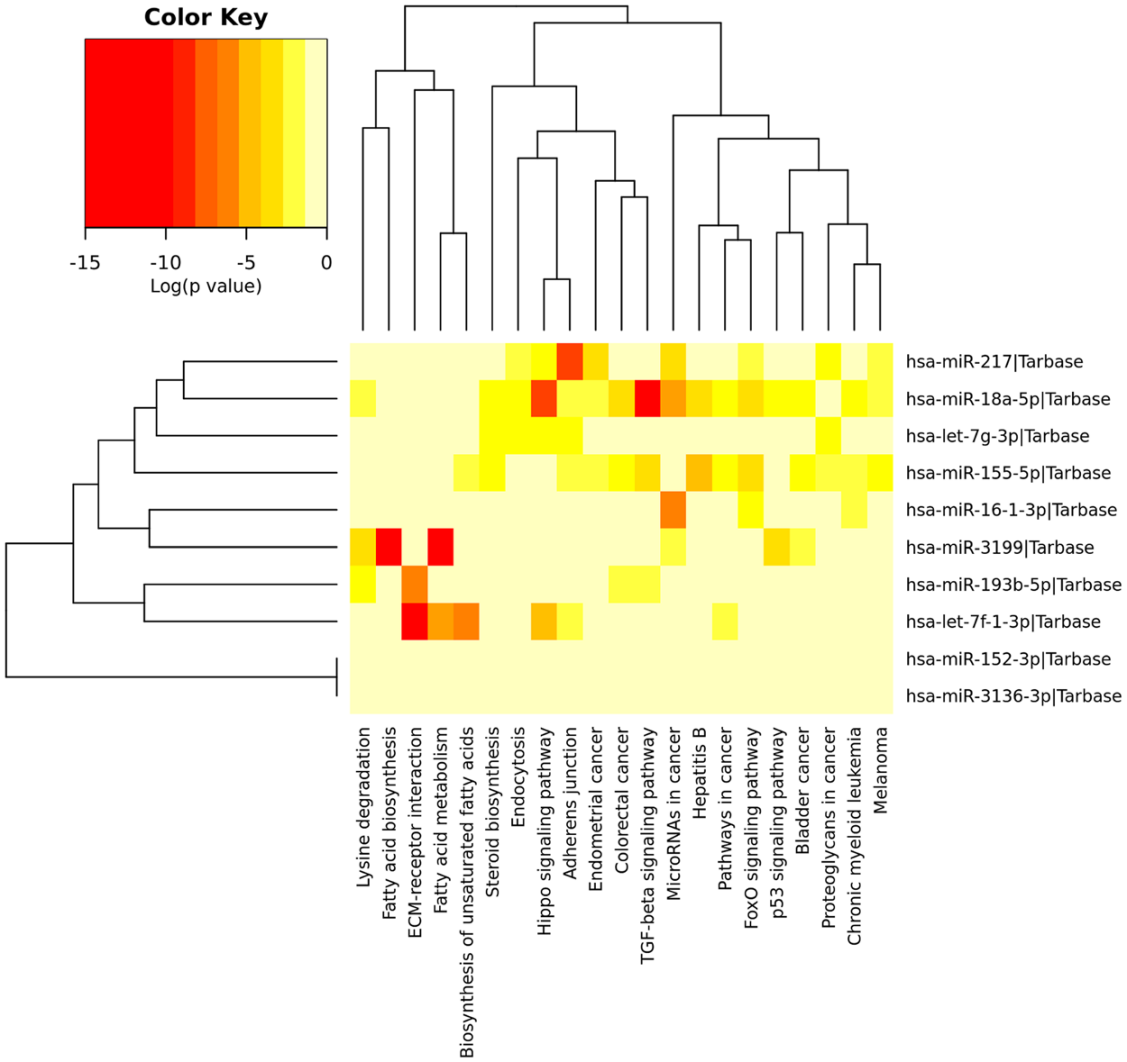
Heat map of the KEGG pathway. Top-10 miRNAs involved in cancer and non-cancer pathways.

The pathway union analysis results showed that hsa-miR-7f-1, hsa-miR-16-1, hsa-miR-217, hsa-miR-18a, hsa-miR-193b, and hsa-miR-3199-1 are highly enriched in specific pathways such as extra cellular biosynthesis of unsaturated fatty acids, miRNAs in cancer pathway, adherence junction, in hippo and TGF-beta signaling pathways, ECM-receptor interaction in fatty acid biosynthesis, and fatty acid metabolism respectively. Additionally, all the top-10 miRNAs are involved in endometrial cancer, colorectal cancer, pathways in cancer, bladder cancer, proteoglycans in cancer, chronic myeloid leukemia, melanoma, hepatitis-B, and lysine degradation, to name a few. The pathway union enrichment analysis is shown in Fig. 3, and KEGG pathway analysis for all the 18 miRNAs are shown in Supplementary Fig. S5.

**Figure 4:**
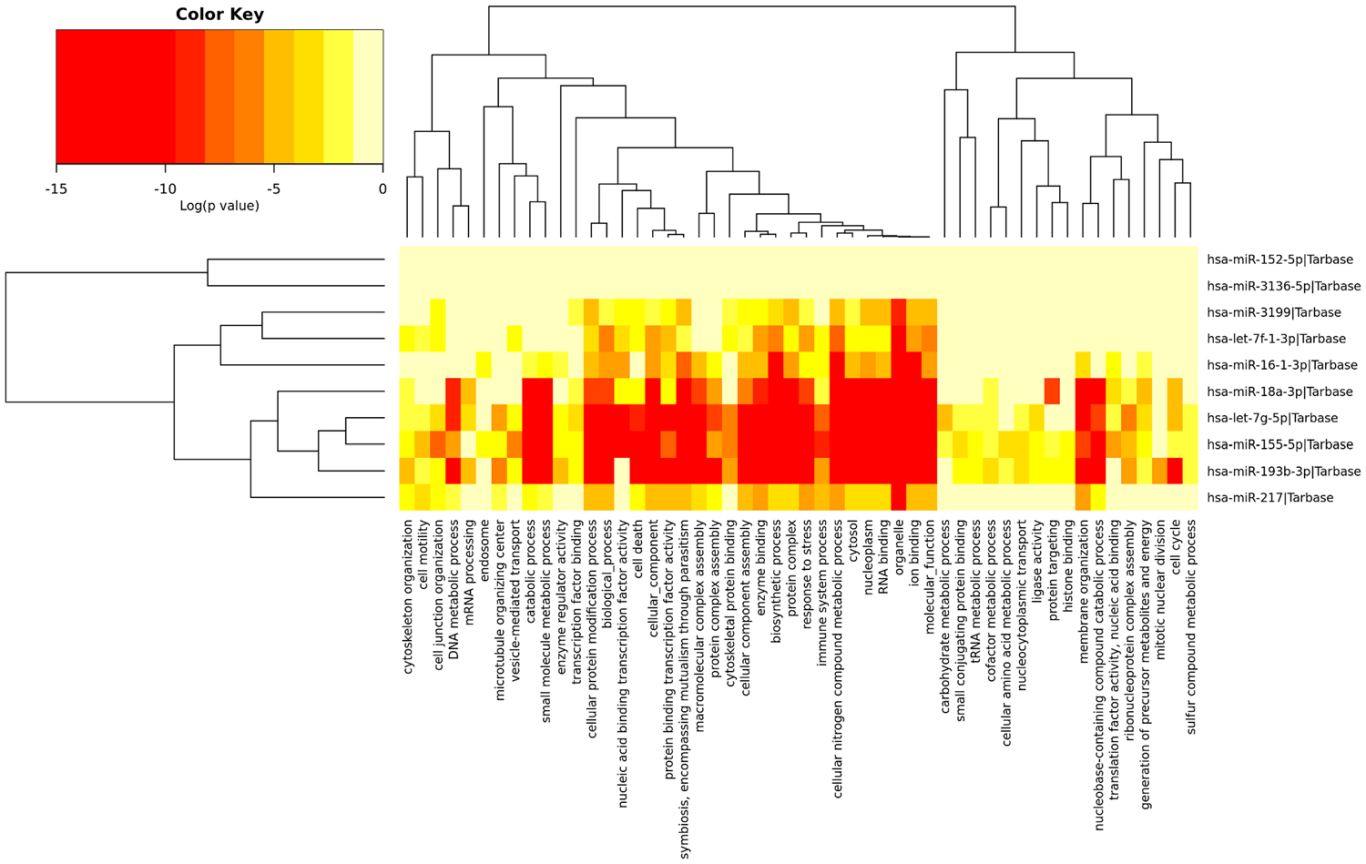
Heat map of the GO term analysis. Top-10 miRNAs involved in cellular component, molecular function and biological pathways in brief.

Secondly, we employed a GO slim to provide a summary of gene ontology annotation for the identified miRNA signature. GO slim annotation results showed that hsa-miR-193, hsa-miR-155, hsa-miR-7g and hsa-miR-18a are highly enriched in specific molecular functions, biological processes and cell components, such as catabolic process, small molecule catabolic process, cellular protein modification process, cell death, cellular component, protein binding transcription factor activity, macro molecular complex assembly, protein complex assembly, cellular component assembly, enzyme binding, biosynthetic process, protein complex, cellular nitrogen compound metabolic process, cytosol, nucleoplasm, RNA binding, organelle, ion binding and molecular functions. Gene ontology enrichment of the top-10 miRNAs are depicted in Fig. 4. All the 18 miRNAs’ GO slim analysis is shown in Supplementary Fig. S6. Detailed process of gene ontology annotation results are shown in Supplementary Table S4.

## Conclusions

Due to the limitation in conventional therapies and diversified nature of diseases, multi-dimensional strategies are needed in cancer therapy. At present there are two strategies for implementing miRNAs as therapeutics in lung cancer. One is to inhibit the function of oncogenic miRNAs and the other is to restore the tumor-suppressor miRNA functions. Promising pre-clinical studies have shown that the therapeutic potentials of miRNAs in cancer treatment by restoring the miRNA functions. For instance, restoration of miR-34 inhibits the tumor growth in animal models^86, 87^. Let-7, miR-31 and miR-16 were proven to have anticancer effect in pre-clinical models^88^. Moreover, the company, miRNA therapeutics, developed a MRX34, a miRNA mimic, which was put into the clinical practice. Thus, identification of an effective miRNA signature can guide therapeutic decision and diagnosis in lung cancer.

Accordingly, we developed a method SVR-LUAD to identify the potential miRNA signature associated with survival time of lung adenocarcinoma patient. We first proposed a support vector regression based method cooperated with an optimal feature selection algorithm IBCGA to identify the miRNA signature associated with survival time in lung adenocarcinoma patients. The proposed survival time estimator identified the 18-miRNA signature out of 332 miRNAs strongly correlated with the survival time of lung cancer patients and obtained a correlation coefficient of 0.90 and mean absolute error of 0.52 year using 10-CV. Further, we ranked miRNAs based on the MED experiment and discussed the top-10 miRNA signatures’ characterization in lung cancer and other major cancers. Among the top-10 miRNA signatures, two miRNAs, hsa-miR-3136 and hsa-miR-3199-1, were previously unreported for the involvement in lung cancer. However, our method has found that these two miRNAs, like the other eight reported miRNAs, are strongly correlated with the survival of lung adenocarcinoma patients. Additionally, gene ontology enrichment annotations and KEGG pathway involvement of these top-10 miRNAs are discussed. The analysis suggests that these two miRNAs might be important subjects for further examination.

Validation of clinical applicability of the miRNA signature is necessary. We hope that the identified miRNA signature will assist in comprehensively understanding their pathway mechanism in lung cancer and improve the therapeutic strategies for the treatment of lung adenocarcinoma patients.

## Materials and Methods

### Dataset

There were 521 patients with lung adenocarcinoma in the TCGA database. We downloaded level-3 miRNA expression data from the TCGA portal that the miRNA profiling was implemented on the Illumina HiSeq. 2000 miRNA sequencing platform. We filtered out the used dataset using the following criteria. We included only the patients who have clinical data and survival information, and excluded the patients with a survival period of less than one month. All patients with their clinical data and survival periods were merged into a single dataset to eliminate duplicate entries. As a result, there were 102 patients with expression profiles of 332 miRNAs along with their clinical data including gender, age, and days until death (survival time).

Another set of 51 patients who are still alive with clinical data and follow-up time was used as an independent test cohort. The 51 patients with lung adenocarcinoma were selected by considering 1) who have tumors after pharmaceutical therapy, 2) vital status (alive), and 3) offer of follow-up days.

### SVR-LUAD

The proposed method SVR-LUAD is an integration approach to combiningνsupport vector regression (ν-SVR) and feature selection algorithm IBCGA. SVR-LUAD is designed to simultaneously identify the miRNA signature and predict the survival time in order to discover the mechanism of the miRNA signature and develop effective therapies of lung adenocarcinoma patients.

### Support vector regression

Support vector machine (SVM) is a state-of-the-art method for solving classification and regression problems. SVM has extensively been used in solving biological problems^89^. SVR is one of the practical modes of SVM. Due to the potential regression ability, SVR has been applied to a wide range of biological issues, such as estimation of survival time in glioblastoma cancer patients^34^, estimation of missing values of microarray data^90^, prediction of gene expression levels^91^, and prediction of siRNA efficacy^92^.

The ν-SVR is a new regression method of SVM which presents good performance depending on the number of support vectors and training error^93^. Given a set of *N* data points, {(x_1_, y_1_), (x_2_, y_2_), …, (x_N_, y_N_)}, where x_i_ $\in$ R^m^ is an input sample (patient) and y_i_ $\in$ R^1^ is a target label. In this study, y_i_ is the survival time. The optimization problem of the ν-SVR can be described as follows.

1 $$min\,\frac{1}{2}{w}^{T}w+b+C(\nu \varepsilon +\,\frac{1}{N}\sum _{i=1}^{N}({\xi }_{i}+{\xi }_{i}^{\ast }))$$

Subject to

2 $$({w}^{T}{\rm{\varnothing }}({x}_{i})+b)-{y}_{i}\le {\rm{\varepsilon }}+{\xi }_{i},$$

3 $${y}_{i}-({w}^{T}{\rm{\varnothing }}({x}_{i})+b)\le {\rm{\varepsilon }}+{\xi }_{i}^{\ast }$$

4 $${\xi }_{i},{\xi }_{i}^{\ast }\ge 0,\,i=1,\ldots .N,{\rm{\varepsilon }}\ge 0$$

where 0 ≤ *v* ≤ 1. *C* is a regularization parameter and b is a constant. The ε-insensitive loss function means that if *w* ^*T*^∅(*xi*) is in the range of y ± ε, no loss is considered. The *y* ^T^ is known as the soft margin where ν is an upper bound on the fraction of margin errors and a lower bound of the fraction of support vectors.

### Fitness function

The fitness function of the IBCGA is the only guide to search for an optimal solution. In this study, the fitness function is to maximize the Pearson’s correlation coefficient (*CC*) of 10-CV as follows:

5 $$max\,CC=\frac{{\sum }_{i=1}^{M}({y}_{i}-\bar{y})\,({z}_{i}-\bar{z})}{\sqrt{{[\sum }_{i=1}^{M}{({y}_{i}-\bar{y})}^{2}]\,[{\sum }_{i=1}^{M}{({z}_{i}-\bar{z})}^{2}]}}$$

where *y*_i_ and *z*_i_ are real and predicted survival time of the *i* ^th^ patient, and $\bar{y}$ and $\bar{z}$ are their corresponding means. *M* is the total number of patients (*M* = 102 in this study). The mean absolute error (*MAE*) is also used for measuring prediction performance:

6 $$MAE=\,\frac{1}{M}\sum _{i=1}^{M}{|{y}_{i}-{z}_{i}|}^{2}$$

### Inheritable bi-objective combinatorial genetic algorithm

SVR-LUAD used the optimal feature selection method IBCGA to identify a small set of *m* informative miRNAs from *n* = 332 miRNAs cooperating with ν-SVR by maximizing estimation accuracy of survival time. The IBCGA uses an intelligent evolutionary algorithm^94^ for solving the large combinatorial optimization problem C(*n*, *m*) to obtain an optimized ν-SVR where *n* is a given large constant and the best value of the variable *m* is not known beforehand. The intelligent evolutionary algorithm uses an orthogonal array crossover with a systematic reasoning ability to reproduce better offspring instead of random recombination in the crossover operation. The intelligent evolutionary algorithm can obtain a good solution *S*_*k*_ to C(*n*, *k*) if *k* is a given constant. The IBCGA can obtain a set of solutions, *S*_*r*_, where *r* = *r*_start_, *r*_start_ + 1, …, *r*_end_ in a single run to efficiently search for a solution *S*_*r*+*1*_ to C(*n*, *r* + *1*) by inheriting a good solution *S*_*r*_ to C(*n*, *r*). The *S*_*m*_ is the best solution among the solutions *S*_*r*_. In this work, the LibSVM package^95^ was used for implementation of ν-SVR.

The chromosome of the IBCGA consists of 332 genes for encoding the 332 miRNAs and three 4-bit genes for encoding the three variables γ, C, andνof theν-SVR. The parameter tuning of IBCGA was same with the previous study^34, 35^. The customized IBCGA for obtaining the *m*-miRNA signature where *r*_start_ ≤ *m*≤ *r*_end_ is described below.

Step 1) (Initialization) Randomly generate an initial population with *N*_*pop*_ individuals. Each individual has *r* 1′s and *n*-*r* 0′s encoded into the *n* binary genes *f*_*i*_, where *r* = *r*_start_.

Step 2) (Evaluation) Evaluate all individuals in the population using the fitness function (2).

Step 3) (Selection) Use a tournament selection method that selects the winner from two randomly selected individuals to form a mating pool.

Step 4) (Crossover) Select *P*_*c*_ · *N*_*pop*_ parents from the mating pool to perform the orthogonal array crossover^94^, where *P*_*c*_ is the crossover probability.

Step 5) (Mutation) A traditional mutation operator is applied to the randomly selected *P*_*m*_ · *N*_*pop*_ individuals except the best individual, where *P*_*m*_ is the mutation probability.

Step 6) (Termination) If the stopping condition of performing *G*_*max*_ generations is satisfied, output the best individual in the population as *S*_*r*_. Otherwise, go to Step 2.

Step 7) (Inheritance) If *r* < *r*_end_, randomly change one bit in the binary genes *f*_*i*_ from 0 to 1 for each individual; increase the number *r* by one, and go to Step 2. Otherwise, stop the algorithm.

Step 8) (Output) Let *m* be equal to the value of *r* that *S*_*r*_ is the best solution in the population. Output the *m* miRNAs and the corresponding ν-SVR model.

### Appearance score

Since the IBCGA is a non-deterministic algorithm that the solutions of multiple runs are not always the same, selection of a robust solution is necessary. SVR-LUAD automatically identifies a robust solution (miRNA signature) from R (R = 30 in this study) independent runs for estimating the survival time of patients with lung adenocarcinoma. The robust set of features (miRNAs) has the highest appearance score obtained using the following procedure.

Step 1: Prepare the training dataset for 10-CV.

Step 2: Perform R independent runs of SVR-LUAD by maximizing CC of 10-CV for obtaining R miRNA signatures. There are ${m}_{t}$ features in the t-th signatures, t = 1, …, R.

Step 3: Appearance score is calculated as follows:

1. Calculate the appearance frequency f(p) for each feature p that ever presents in the R sets of miRNAs.
2. Calculate the score S_t_, t = 1, …, R where p_i_ is the i-th feature in the t-th solution:

7 $${S}_{t}=\sum _{i=1}^{{m}_{t}}\,f({p}_{i})/{m}_{t}$$

Step 4: Output the t-th feature set with the highest appearance score S_t_.

### Multiple regression analysis

We employed the Multiple linear regression method^96^ to estimate the survival time in lung adenocarcinoma patients. The Multiple linear regression method is formulated as

8 $${y}_{i}={\beta }_{0}+\,{\beta }_{1}{x}_{i1}+\,{\beta }_{2}{x}_{i2}+\cdots +{\beta }_{m}{x}_{im}+\,\varepsilon ,$$

where *y*_*i*_ is a dependent variable (survival time of the *i*-th patient in this study); *x*_*i1*_, *x*_*i2*_, …, and *x*_*i*m_ are independent variables (miRNA expression); *β*_*0*_ is a regression constant; *β*_*1*_ *, β*_*2*_, *…*, and *β*_*m*_ are the regression coefficients; *m* is the number of terms in the model, and ε is the error term. In this study, *m* is the number of selected miRNAs. A stepwise feature addition method was used for feature selection^97^.

### Elastic net

Elastic net is a regularization with an automatic feature selection technique^98^, which is a combination of ridge regression^99^ and least absolute shrinkage and selection operator (LASSO)^100^. The objective function of the Elastic net method using 10-CV is formulated as follows:

9 $$Min{}_{{\beta }_{0},\beta }\,(\frac{1}{2M}\sum _{i=1}^{M}{({y}_{i}-{\beta }_{0}-{x}_{i}^{T}\beta )}^{2}+\,\lambda {P}_{\alpha }(\beta ))$$

where *y*_i_ is the sample response (survival time) at observation *i* (patient); *x*_*i*_ $\in$ R^*m*^ is the vector of *m* miRNA expression values for the *i-*th observation, λ is a regularization parameter, *β*_*0*_ and *β* are regression coefficients, and *M* is the total number of observations.

### KEGG pathway and Gene ontology annotation analysis

We used DIANA-mirpath web-based server to analyze the miRNA profiles^101^. The DIANA-Tarbase algorithm provided the predicted miRNAs targets for the pathway analysis. We used fisher’s exact test for enrichment analysis with a threshold p-value 0.05. In order to estimate the specificity of results, we performed the pathway analysis for all identified miRNAs.

We employed gene ontology annotations in order to identify miRNAs belonging to specific GO categories based on the experimental findings using the DIANA-mirpath webserver^101^. This webserver uses predicted miRNA targets obtained from the DIANA-microT-CDS algorithm. We used a hypergeometric distribution method for enrichment analysis.

## Electronic supplementary material

Supplementary Information
