# Supplementary material for: Identifying the miRNA signature associated with survival time in patients with lung adenocarcinoma using miRNA expression profiles
Source: Sci Rep. 2017 Aug 8;7:7507. doi: 10.1038/s41598-017-07739-y (PMC5548864; doi:10.1038/s41598-017-07739-y)
Supplement: Supplementary file 1 — Supplementary Information [file 41598_2017_7739_MOESM1_ESM.docx]

**Identifying the miRNA signature associated with survival time in patients with lung adenocarcinoma using miRNA expression profiles**

**Yerukala Sathipati Srinivasulu^1^ and Shinn-Ying Ho^1, 2*^**

^1^Institute of Bioinformatics and Systems Biology, National Chiao Tung University, Hsinchu, Taiwan

^2^Department of Biological Science and Technology, National Chiao Tung University, Hsinchu, Taiwan

*Corresponding author

Email address:

SYH: [syho@mail.nctu.edu.tw](mailto:syho@mail.nctu.edu.tw)

# Supplementary Figures


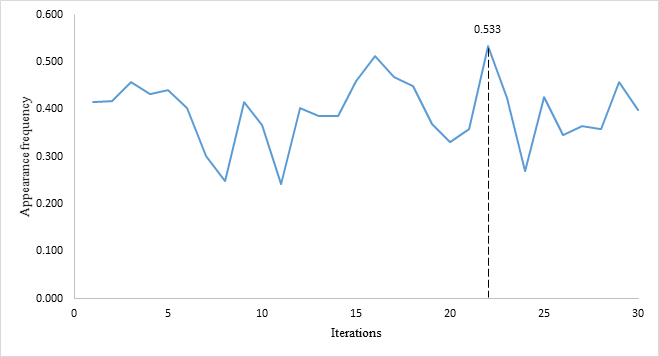


**Supplementary Figure S1. Robust feature set selection**. Appearance scores of individual runs shown on Y-axis and number of iterations on the X-axis.


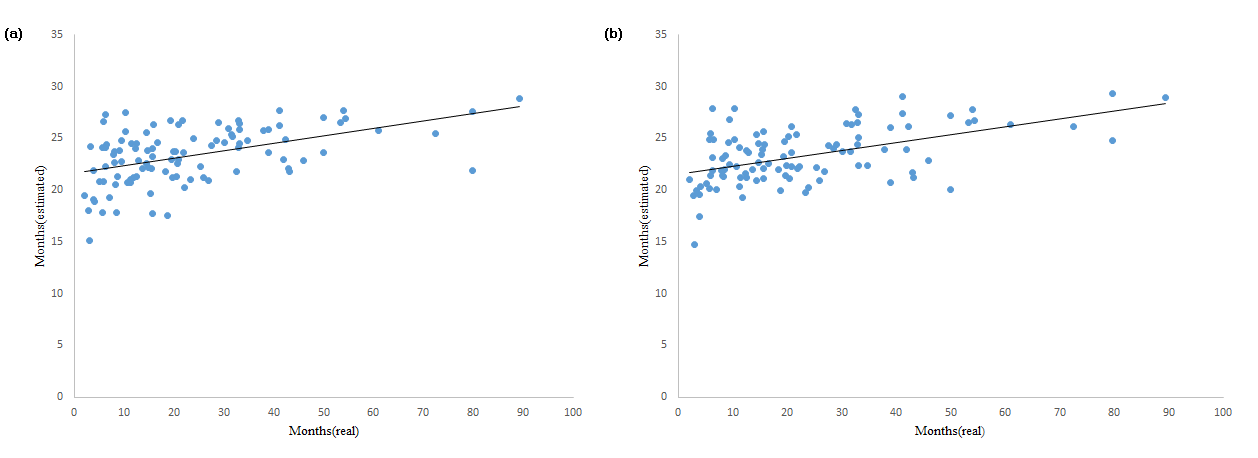


**Supplementary Figure S2.** X-axis refers to real survival time and Y-axis refers to estimated survival time. (a) Prediction performance of Lasso with a correlation coefficient of 0.48. (b). Prediction performance of Ridge regression with a correlation coefficient of 0.51.


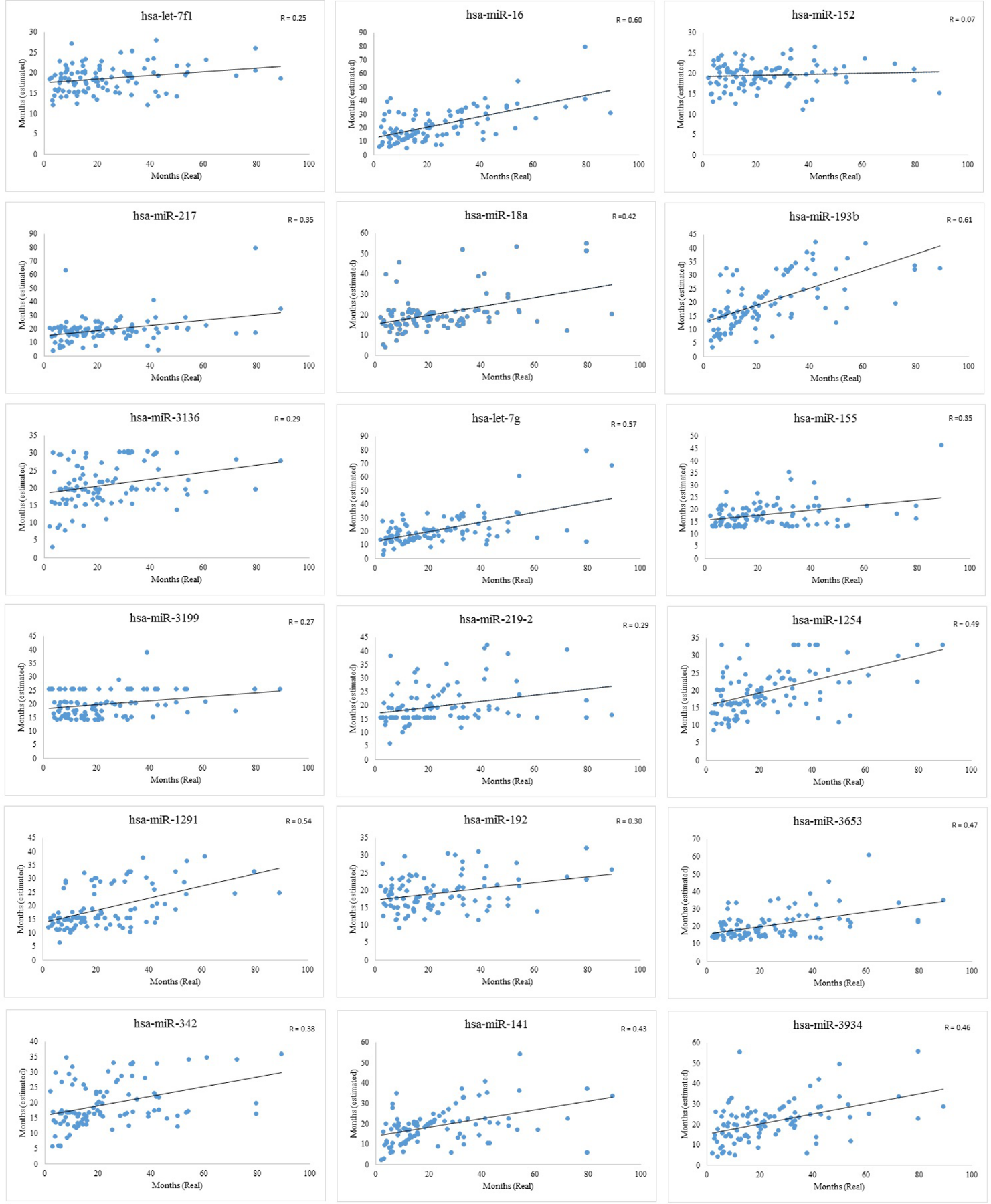


**Supplementary Figure S3.** **Individual effect of miRNAs on survival prediction**.


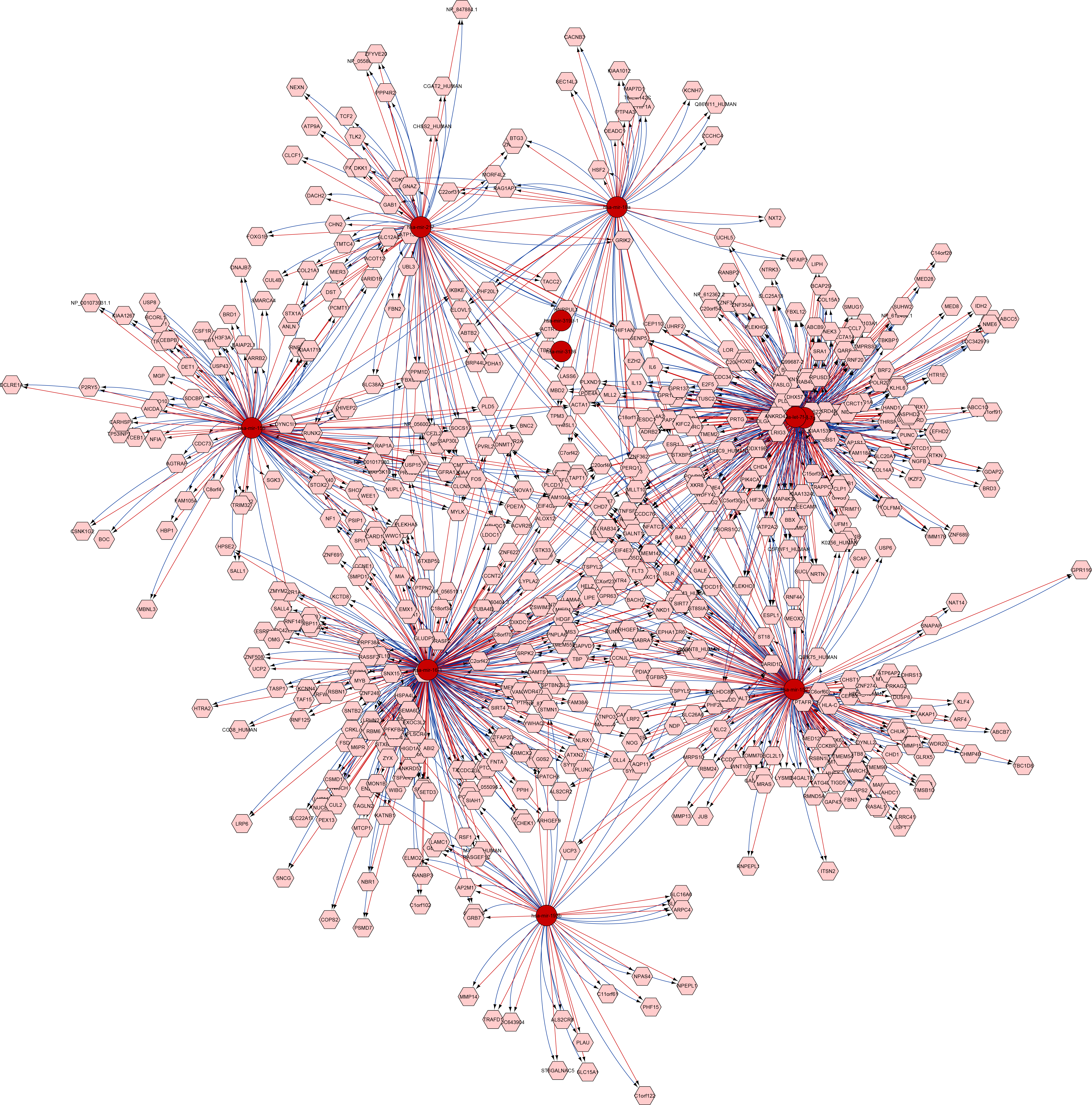


**Supplementary Figure S4. MiRNA and gene target network**. The top-10 miRNAs and corresponding target genes predicted using MicroCosm and TargetScan. In this network microRNAs and target genes are defined as red circles and pink rounded hexagons respectively. The predicted microRNA-Target interactions are visualized in blue (Target scan: 762 target interactions) and in red color (MicroCosm: 762 target interactions). Overlap threshold was set to 2 between two regulatory interaction networks. Picture was drawn using CyTargetLinker.


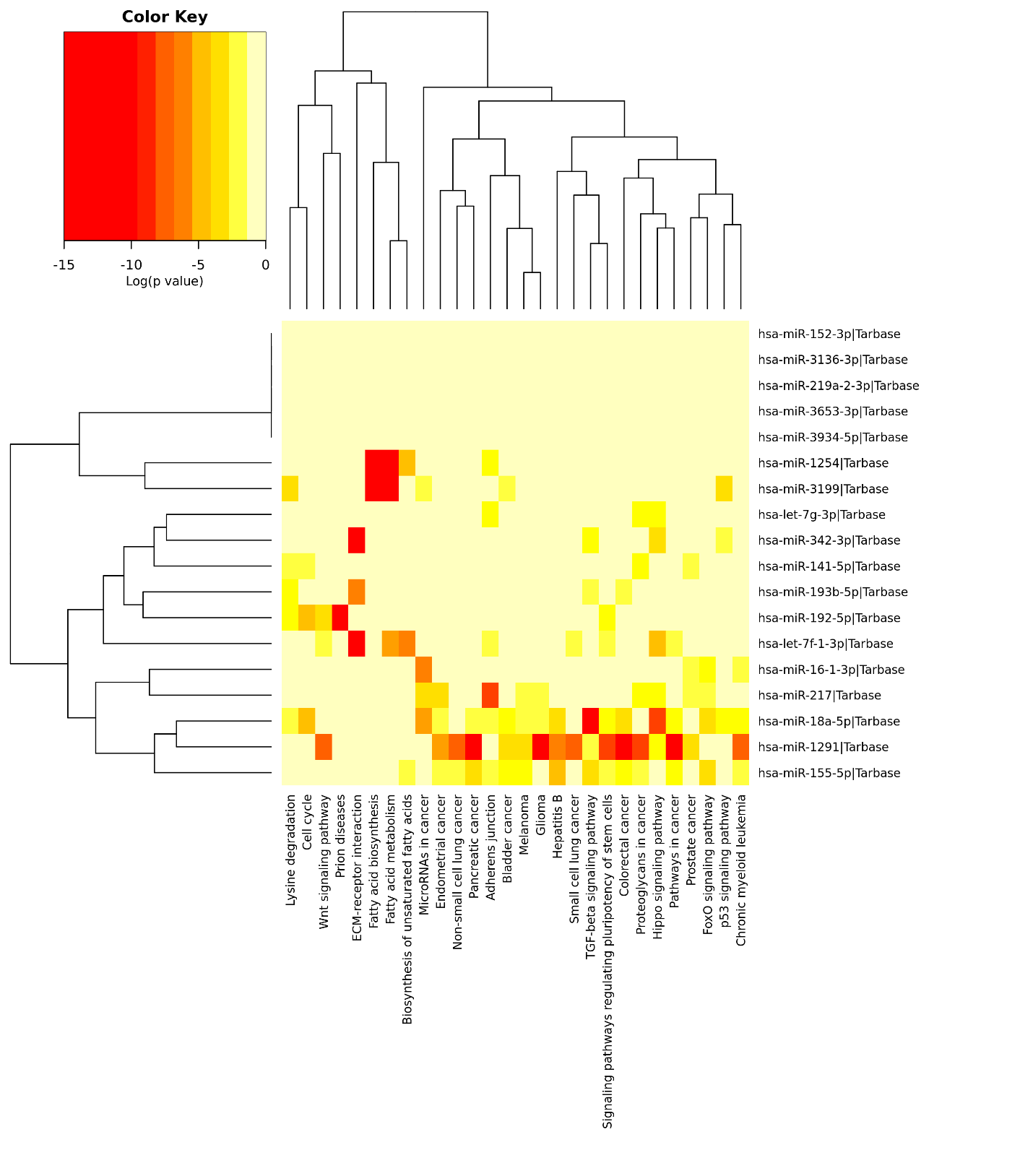


**Supplementary Figure S5. Heat map of the KEGG pathway**. The 18-miRNA signature involved in cancer and non-cancer pathways.


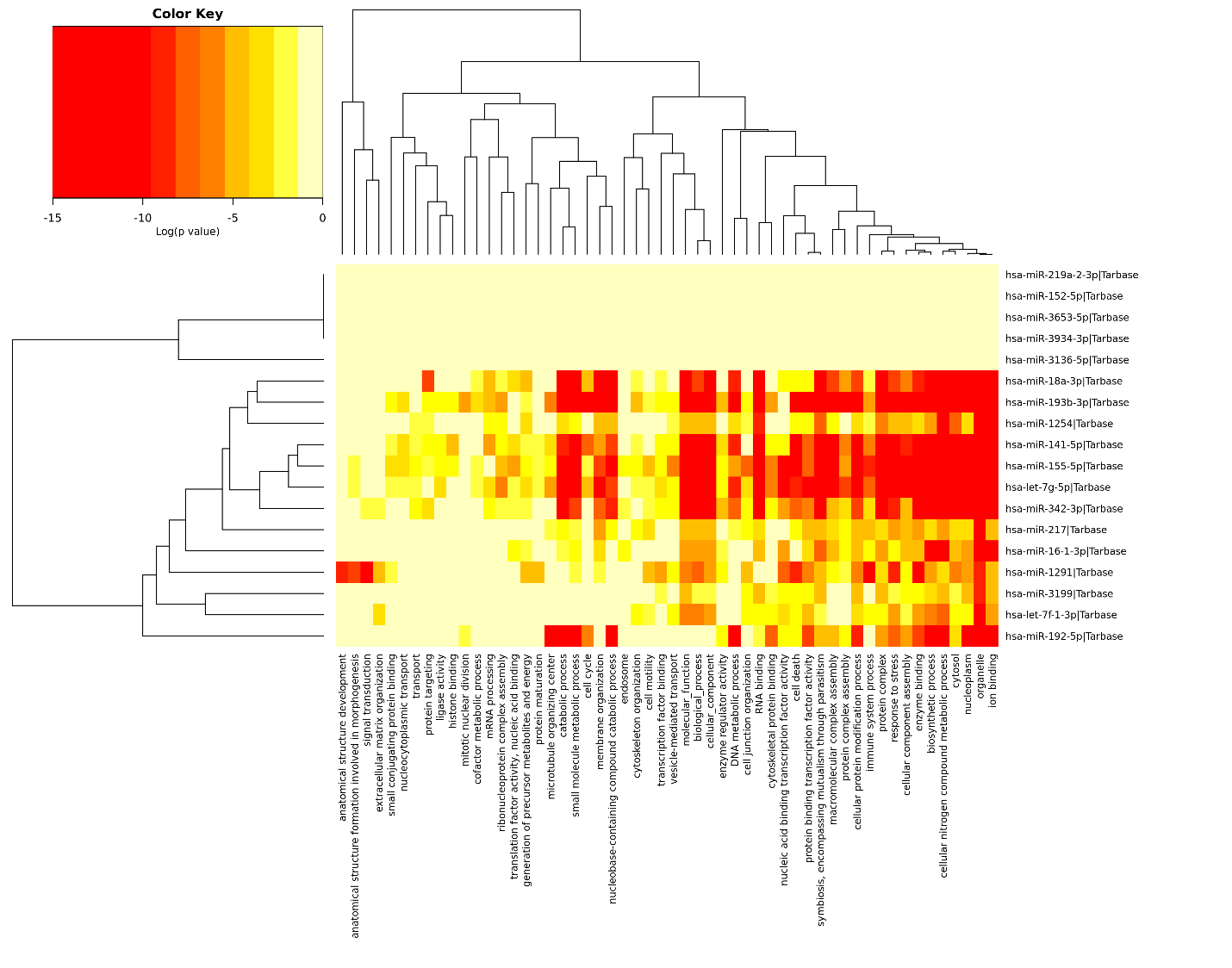


**Supplementary Figure S6. Heat map of the GO term analysis**. The 18-miRNAs signature involved in cellular component, molecular function, and biological pathways in brief

# Supplementary Tables

**Supplementary Table S1. Appearance scores**. Correlation coefficient (CC), mean absolute error (MAE) and appearance score for each iteration

| **Runs** | **MAE** | **CC** | **Features selected** | **APP** |
| --- | --- | --- | --- | --- |
| 1 | 0.535 | 0.894 | 22 | 0.415 |
| 2 | 0.526 | 0.902 | 28 | 0.418 |
| 3 | 0.582 | 0.871 | 20 | 0.458 |
| 4 | 0.518 | 0.901 | 27 | 0.432 |
| 5 | 0.543 | 0.902 | 28 | 0.440 |
| 6 | 0.564 | 0.896 | 30 | 0.403 |
| 7 | 0.564 | 0.885 | 29 | 0.302 |
| 8 | 0.675 | 0.859 | 39 | 0.250 |
| 9 | 0.544 | 0.880 | 22 | 0.415 |
| 10 | 0.640 | 0.857 | 16 | 0.367 |
| 11 | 0.585 | 0.887 | 28 | 0.242 |
| 12 | 0.525 | 0.885 | 28 | 0.404 |
| 13 | 0.593 | 0.876 | 30 | 0.387 |
| 14 | 0.567 | 0.881 | 30 | 0.386 |
| 15 | 0.533 | 0.896 | 23 | 0.459 |
| 16 | 0.572 | 0.887 | 18 | 0.513 |
| 17 | 0.580 | 0.877 | 23 | 0.468 |
| 18 | 0.554 | 0.887 | 20 | 0.448 |
| 19 | 0.586 | 0.874 | 29 | 0.370 |
| 20 | 0.562 | 0.887 | 29 | 0.331 |
| 21 | 0.574 | 0.888 | 26 | 0.359 |
| 22 | 0.522 | 0.900 | 18 | 0.533 |
| 23 | 0.553 | 0.877 | 17 | 0.424 |
| 24 | 0.610 | 0.871 | 31 | 0.270 |
| 25 | 0.553 | 0.885 | 19 | 0.426 |
| 26 | 0.564 | 0.884 | 28 | 0.346 |
| 27 | 0.604 | 0.872 | 26 | 0.364 |
| 28 | 0.610 | 0.872 | 17 | 0.359 |
| 29 | 0.551 | 0.885 | 18 | 0.457 |
| 30 | 0.547 | 0.885 | 22 | 0.398 |

**Supplementary Table S2. Top-10 miRNAs and experimentally validated target genes**

| **Rank** | **miRNAs** | **Experimentally validated**  **Target Gene** | **Experimental Methods** | **Reference** |
| --- | --- | --- | --- | --- |
| 1 | hsa-let-7f-1 | CSN1, CSN6  CSN8, | Western blot | 1 |
|  |  | PRDM1 | Immunohistochemistry , Luciferase reporter assay , qPCR , Western blot | 2 |
|  |  | HMGA2 | Immunoprecipitation | 3-5 |
|  |  | TG, CCND1, CDKN1A, MYC, TITF1 | qPCR | 6 |
| 2 | hsa-miR-16-1 | CCD1 | qPCR, Reporter assay, Western blot | 7 |
|  |  | CCNE1 | Reporter assay | 8 |
|  |  | HGF | Immunoprecipitation | 9 |
|  |  | ZYX | qPCR | 10 |
| 3 | hsa-miR-152 | HLA-G, TGFA | Reporter assay | 11,12 |
|  |  | DNMT1 | Reporter assay, Western blot, qPCR | 13 |
|  |  | IRS1, IGF1R, | Reporter assay, Western blot | 14 |
| 4 | hsa-miR-217 | SIRT1, ROBO1, DACH1, FOXO3, GPC5 | Reporter assay, Western blot, qPCR | 15-19 |
|  |  | EZH2 | Immunohistochemistry, Luciferase reporter assay, qPCR , Western blot | 20 |
|  |  | E2F3 | Immunohistochemistry , Luciferase reporter assay , Western blot | 21 |
|  |  | NR4A2, TRAPPC2P1, PPM1D, FHIT, KRAS, SMAD7 | Reporter assay | 22-27 |
| 5 | hsa-miR-18a | KRAS, ESR1, SMAD4, HSF2, ATM | Reporter assay, Western blot, qPCR | 28-33 |
|  |  | PTEN | Immunohistochemistry , Luciferase reporter assay , qPCR , Western blot | 34 |
|  |  | CTGF, NR3C1 | Reporter assay, Western blot, | 35,36 |
|  |  | NEDD9, CDK19, | Western blot, qPCR | 37 |
|  |  | NACO3, SMAD3 | Western blot | 29,38 |
| 6 | hsa-miR-193b | CCND1, PLAU, MCL1, ETS1, | Reporter assay, Western blot, qPCR | 39-42 |
|  |  | PRAP1 | Reporter assay, Western blot, | 40 |
| 7 | hsa-miR-3136 | SOCS5, IBA57, RAC2, DAZAP2, VEZF1, HIST1H2BG, CALM2, AGFG1, NHLRC3 | Immunoprecipitation | 43 |
| 8 | hsa-let-7g | COL1A2, MYC, HMGA2, IGF2BP1, CDKN2A, BCL2L1, | Reporter assay, Western blot, qPCR | 44-48 |
|  |  | AGO1 | Immunohistochemistry , Luciferase reporter assay , Northern blot , qPCR , Western blot | 49 |
|  |  | BMI1 | Western blot, qPCR | 45 |
|  |  | GAB2, FN1 | Reporter assay, Western blot | 50 |
| 9 | hsa-miR-155 | MEIS1, TAB2, SOCS1, INPP5D, SMAD5, HIVEP2, ZNF652 | Reporter assay, Western blot, qPCR | 51-57 |
|  |  | MLH1, JMJD1A, BACH1 | Immunohistochemistry , Luciferase reporter assay , qPCR , Western blot | 58,59 |
|  |  | MSH6, MSH2 | Reporter assay, Western blot | 60 |
| 10 | hsa-miR-3199-1 | CDK16, SOX4, ACO2, SESN2, UBE2Z, SETD1A, DGCR2, BCl11A, SPSB1, PEG10, PABPN1, SCD, IPO7LARP1 | Immunoprecipitation | 3 |

**Supplementary Table S3.** The top-10 miRNAs and their target gene involved in the KEGG pathway.

| KEGG pathway | miRNAs | genes | p-value |
| --- | --- | --- | --- |
| Fatty acid biosynthesis | 1 | 1 | 0 |
| Fatty acid metabolism | 2 | 4 | 0 |
| Hippo signaling pathway | 4 | 36 | 9.15E-11 |
| ECM-receptor interaction | 2 | 9 | 1.15E-10 |
| TGF-beta signaling pathway | 3 | 19 | 5.92E-10 |
| MicroRNAs in cancer | 4 | 33 | 8.67E-10 |
| Adherens junction | 5 | 29 | 4.12E-08 |
| FoxO signaling pathway | 4 | 51 | 0.000115 |
| Hepatitis B | 2 | 36 | 0.000255 |
| Colorectal cancer | 3 | 27 | 0.000405 |
| Pathways in cancer | 3 | 86 | 0.000878 |
| p53 signaling pathway | 2 | 16 | 0.002546 |
| Proteoglycans in cancer | 3 | 35 | 0.00312 |
| Lysine degradation | 3 | 12 | 0.005688 |
| Biosynthesis of unsaturated fatty acids | 2 | 6 | 0.007267 |
| Endocytosis | 3 | 33 | 0.011366 |
| Bladder cancer | 3 | 18 | 0.016113 |
| Melanoma | 3 | 23 | 0.023461 |
| Chronic myeloid leukemia | 3 | 26 | 0.024667 |
| Steroid biosynthesis | 3 | 7 | 0.033863 |
| Endometrial cancer | 3 | 17 | 0.035723 |
| Prostate cancer | 2 | 13 | 0.063553 |
| Fatty acid elongation | 2 | 5 | 0.101342 |
| Signaling pathways regulating pluripotency of stem cells | 3 | 36 | 0.112098 |
| Arrhythmogenic right ventricular cardiomyopathy (ARVC) | 2 | 11 | 0.115821 |
| Pancreatic cancer | 2 | 25 | 0.12562 |
| Bacterial invasion of epithelial cells | 1 | 3 | 0.140526 |
| Glioma | 2 | 12 | 0.148271 |
| Thyroid hormone signaling pathway | 2 | 18 | 0.155163 |
| Cell cycle | 1 | 23 | 0.161484 |
| Regulation of actin cytoskeleton | 2 | 14 | 0.168555 |
| Transcriptional misregulation in cancer | 1 | 12 | 0.177386 |
| Viral carcinogenesis | 1 | 11 | 0.203299 |
| Small cell lung cancer | 1 | 7 | 0.275649 |
| HTLV-I infection | 1 | 28 | 0.525413 |
| Salmonella infection | 1 | 4 | 0.574251 |
| Tight junction | 1 | 10 | 0.618494 |
| TNF signaling pathway | 1 | 22 | 0.647985 |
| Leukocyte transendothelial migration | 1 | 8 | 0.677508 |
| Epithelial cell signaling in Helicobacter pylori infection | 1 | 9 | 0.704755 |
| Pathogenic Escherichia coli infection | 1 | 4 | 0.705634 |
| Non-small cell lung cancer | 1 | 11 | 0.747996 |
| Hypertrophic cardiomyopathy (HCM) | 1 | 3 | 0.780237 |
| Apoptosis | 1 | 19 | 0.803839 |
| Inflammatory bowel disease (IBD) | 1 | 13 | 0.80797 |
| Protein processing in endoplasmic reticulum | 1 | 21 | 0.813255 |
| mTOR signaling pathway | 1 | 6 | 0.82245 |
| NF-kappa B signaling pathway | 1 | 16 | 0.849745 |
| Viral myocarditis | 1 | 5 | 0.863198 |
| Malaria | 1 | 14 | 0.877467 |
| Dilated cardiomyopathy | 1 | 3 | 0.904181 |
| Wnt signaling pathway | 1 | 7 | 0.914617 |
| Estrogen signaling pathway | 1 | 7 | 0.944191 |
| Chagas disease (American trypanosomiasis) | 1 | 18 | 0.957569 |
| Spliceosome | 1 | 14 | 0.961977 |
| Phagosome | 1 | 10 | 0.980561 |
| Ubiquitin mediated proteolysis | 1 | 17 | 0.988498 |
| Vitamin B6 metabolism | 1 | 2 | 0.991946 |
| Phosphatidylinositol signaling system | 1 | 3 | 0.992693 |
| D-Glutamine and D-glutamate metabolism | 1 | 1 | 0.998722 |

**Supplementary Table S4.** Gene ontology analysis of the identified miRNA signature.

| **GO Category** | **MiRNAs** | **Genes** | **p-value** |
| --- | --- | --- | --- |
| organelle | 9 | 2951 | 0 |
| cellular nitrogen compound metabolic process | 9 | 1514 | 9.49E-188 |
| biosynthetic process | 9 | 1231 | 1.10E-120 |
| ion binding | 9 | 1634 | 7.10E-111 |
| protein complex | 9 | 1159 | 6.20E-84 |
| cellular protein modification process | 9 | 746 | 2.81E-82 |
| nucleoplasm | 9 | 477 | 2.42E-80 |
| cytosol | 9 | 886 | 4.01E-76 |
| symbiosis, encompassing mutualism through parasitism | 8 | 237 | 1.67E-70 |
| molecular_function | 9 | 4015 | 5.16E-65 |
| small molecule metabolic process | 9 | 700 | 3.32E-64 |
| catabolic process | 9 | 613 | 4.45E-62 |
| enzyme binding | 9 | 458 | 3.28E-61 |
| cellular component assembly | 9 | 447 | 2.28E-56 |
| cellular_component | 9 | 4028 | 2.69E-55 |
| RNA binding | 9 | 609 | 6.97E-53 |
| macromolecular complex assembly | 9 | 329 | 8.77E-53 |
| biological_process | 9 | 3916 | 2.71E-50 |
| response to stress | 9 | 672 | 4.49E-49 |
| membrane organization | 9 | 234 | 1.12E-47 |
| protein binding transcription factor activity | 8 | 189 | 5.34E-38 |
| nucleobase-containing compound catabolic process | 9 | 296 | 3.25E-37 |
| DNA metabolic process | 8 | 268 | 1.53E-33 |
| protein complex assembly | 9 | 258 | 6.24E-32 |
| cell death | 8 | 286 | 3.56E-28 |
| nucleic acid binding transcription factor activity | 8 | 270 | 6.13E-20 |
| immune system process | 9 | 418 | 5.00E-19 |
| cytoskeletal protein binding | 9 | 220 | 1.29E-17 |
| ribonucleoprotein complex assembly | 7 | 63 | 1.63E-14 |
| cell cycle | 8 | 272 | 8.53E-14 |
| cell junction organization | 8 | 58 | 1.57E-12 |
| vesicle-mediated transport | 9 | 271 | 4.62E-11 |
| microtubule organizing center | 9 | 134 | 1.01E-10 |
| generation of precursor metabolites and energy | 8 | 97 | 1.31E-10 |
| enzyme regulator activity | 9 | 206 | 5.57E-10 |
| protein targeting | 8 | 88 | 4.25E-09 |
| mRNA processing | 8 | 150 | 7.24E-08 |
| translation factor activity, nucleic acid binding | 7 | 42 | 1.41E-07 |
| cytoskeleton organization | 8 | 170 | 3.16E-07 |
| cell motility | 8 | 142 | 4.58E-07 |
| cofactor metabolic process | 9 | 69 | 5.05E-07 |
| nucleocytoplasmic transport | 9 | 99 | 8.81E-07 |
| sulfur compound metabolic process | 7 | 73 | 3.28E-06 |
| small conjugating protein binding | 6 | 31 | 8.85E-06 |
| ligase activity | 8 | 160 | 9.91E-06 |
| tRNA metabolic process | 5 | 50 | 1.40E-05 |
| transcription factor binding | 8 | 160 | 2.03E-05 |
| cellular amino acid metabolic process | 9 | 104 | 4.45E-05 |
| lipid metabolic process | 8 | 276 | 0.0001709 |
| homeostatic process | 8 | 180 | 0.0002043 |
| cytoskeleton-dependent intracellular transport | 8 | 31 | 0.0002875 |
| mitotic nuclear division | 8 | 110 | 0.0004303 |
| vacuole | 7 | 74 | 0.0004821 |
| histone binding | 7 | 51 | 0.0009153 |
| protein maturation | 8 | 48 | 0.0027121 |
| carbohydrate metabolic process | 9 | 215 | 0.0031062 |
| transport | 9 | 850 | 0.0063251 |
| protein binding, bridging | 8 | 37 | 0.0089695 |
| nuclease activity | 8 | 59 | 0.0141005 |
| endosome | 8 | 144 | 0.0177787 |
| lipid binding | 8 | 130 | 0.027498 |
| plasma membrane organization | 7 | 30 | 0.0322489 |
| extracellular matrix organization | 8 | 74 | 0.0473292 |
| anatomical structure formation involved in morphogenesis | 8 | 124 | 0.0473292 |
| ATPase activity | 7 | 138 | 0.049199 |

# References

1 Leppert, U., Henke, W., Huang, X., Muller, J. M. & Dubiel, W. Post-transcriptional fine-tuning of COP9 signalosome subunit biosynthesis is regulated by the c-Myc/Lin28B/let-7 pathway. *Journal of molecular biology* **409**, 710-721, doi:10.1016/j.jmb.2011.04.041 (2011).

2 Nie, K. *et al.* Epigenetic down-regulation of the tumor suppressor gene PRDM1/Blimp-1 in diffuse large B cell lymphomas: a potential role of the microRNA let-7. *The American journal of pathology* **177**, 1470-1479, doi:10.2353/ajpath.2010.091291 (2010).

3 Karginov, F. V. & Hannon, G. J. Remodeling of Ago2-mRNA interactions upon cellular stress reflects miRNA complementarity and correlates with altered translation rates. *Genes & development* **27**, 1624-1632, doi:10.1101/gad.215939.113 (2013).

4 Balakrishnan, I. *et al.* Genome-wide analysis of miRNA-mRNA interactions in marrow stromal cells. *Stem cells (Dayton, Ohio)* **32**, 662-673, doi:10.1002/stem.1531 (2014).

5 Kishore, S. *et al.* A quantitative analysis of CLIP methods for identifying binding sites of RNA-binding proteins. *Nature methods* **8**, 559-564, doi:10.1038/nmeth.1608 (2011).

6 Ricarte-Filho, J. C. *et al.* Effects of let-7 microRNA on Cell Growth and Differentiation of Papillary Thyroid Cancer. *Translational oncology* **2**, 236-241 (2009).

7 Chen, R. W. *et al.* Truncation in CCND1 mRNA alters miR-16-1 regulation in mantle cell lymphoma. *Blood* **112**, 822-829, doi:10.1182/blood-2008-03-142182 (2008).

8 Wang, F., Fu, X. D., Zhou, Y. & Zhang, Y. Down-regulation of the cyclin E1 oncogene expression by microRNA-16-1 induces cell cycle arrest in human cancer cells. *BMB reports* **42**, 725-730 (2009).

9 Kameswaran, V. *et al.* Epigenetic regulation of the DLK1-MEG3 microRNA cluster in human type 2 diabetic islets. *Cell metabolism* **19**, 135-145, doi:10.1016/j.cmet.2013.11.016 (2014).

10 Li, X. *et al.* MiR-16-1 plays a role in reducing migration and invasion of glioma cells. *Anatomical record (Hoboken, N.J. : 2007)* **296**, 427-432, doi:10.1002/ar.22626 (2013).

11 Tan, Z. *et al.* Allele-specific targeting of microRNAs to HLA-G and risk of asthma. *American journal of human genetics* **81**, 829-834, doi:10.1086/521200 (2007).

12 Zhu, C. *et al.* miR-152 controls migration and invasive potential by targeting TGFalpha in prostate cancer cell lines. *The Prostate* **73**, 1082-1089, doi:10.1002/pros.22656 (2013).

13 Ji, W. *et al.* MicroRNA-152 targets DNA methyltransferase 1 in NiS-transformed cells via a feedback mechanism. *Carcinogenesis* **34**, 446-453, doi:10.1093/carcin/bgs343 (2013).

14 Xu, Q. *et al.* A regulatory circuit of miR-148a/152 and DNMT1 in modulating cell transformation and tumor angiogenesis through IGF-IR and IRS1. *Journal of molecular cell biology* **5**, 3-13, doi:10.1093/jmcb/mjs049 (2013).

15 Deng, S. *et al.* Chronic pancreatitis and pancreatic cancer demonstrate active epithelial-mesenchymal transition profile, regulated by miR-217-SIRT1 pathway. *Cancer letters* **355**, 184-191, doi:10.1016/j.canlet.2014.08.007 (2014).

16 Tie, J. *et al.* MiR-218 inhibits invasion and metastasis of gastric cancer by targeting the Robo1 receptor. *PLoS genetics* **6**, e1000879, doi:10.1371/journal.pgen.1000879 (2010).

17 Zhang, Q., Yuan, Y., Cui, J., Xiao, T. & Jiang, D. MiR-217 Promotes Tumor Proliferation in Breast Cancer via Targeting DACH1. *J Cancer* **6**, 184-191, doi:10.7150/jca.10822 (2015).

18 Zhang, S. *et al.* MicroRNA-217 promotes angiogenesis of human cytomegalovirus-infected endothelial cells through downregulation of SIRT1 and FOXO3A. *PloS one* **8**, e83620, doi:10.1371/journal.pone.0083620 (2013).

19 Wang, H. *et al.* The MicroRNA-217 Functions as a Potential Tumor Suppressor in Gastric Cancer by Targeting GPC5. *PloS one* **10**, e0125474, doi:10.1371/journal.pone.0125474 (2015).

20 Chen, D. L. *et al.* microRNA-217 inhibits tumor progression and metastasis by downregulating EZH2 and predicts favorable prognosis in gastric cancer. *Oncotarget* **6**, 10868-10879, doi:10.18632/oncotarget.3451 (2015).

21 Su, J., Wang, Q., Liu, Y. & Zhong, M. miR-217 inhibits invasion of hepatocellular carcinoma cells through direct suppression of E2F3. *Molecular and cellular biochemistry* **392**, 289-296, doi:10.1007/s11010-014-2039-x (2014).

22 Tsai, Z. Y. *et al.* Identification of microRNAs regulated by activin A in human embryonic stem cells. *Journal of cellular biochemistry* **109**, 93-102, doi:10.1002/jcb.22385 (2010).

23 Wu, F. *et al.* MicroRNAs are differentially expressed in ulcerative colitis and alter expression of macrophage inflammatory peptide-2 alpha. *Gastroenterology* **135**, 1624-1635.e1624, doi:10.1053/j.gastro.2008.07.068 (2008).

24 Zhang, X. *et al.* Oncogenic Wip1 phosphatase is inhibited by miR-16 in the DNA damage signaling pathway. *Cancer research* **70**, 7176-7186, doi:10.1158/0008-5472.can-10-0697 (2010).

25 Lin, Y. X. *et al.* microRNA-143 protects cells from DNA damage-induced killing by downregulating FHIT expression. *Cancer biotherapy & radiopharmaceuticals* **26**, 365-372, doi:10.1089/cbr.2010.0914 (2011).

26 Zhao, W. G. *et al.* The miR-217 microRNA functions as a potential tumor suppressor in pancreatic ductal adenocarcinoma by targeting KRAS. *Carcinogenesis* **31**, 1726-1733, doi:10.1093/carcin/bgq160 (2010).

27 Xia, H., Ooi, L. L. & Hui, K. M. MicroRNA-216a/217-induced epithelial-mesenchymal transition targets PTEN and SMAD7 to promote drug resistance and recurrence of liver cancer. *Hepatology* **58**, 629-641, doi:10.1002/hep.26369 (2013).

28 Tsang, W. P. & Kwok, T. T. The miR-18a* microRNA functions as a potential tumor suppressor by targeting on K-Ras. *Carcinogenesis* **30**, 953-959, doi:10.1093/carcin/bgp094 (2009).

29 Castellano, L. *et al.* The estrogen receptor-alpha-induced microRNA signature regulates itself and its transcriptional response. *Proceedings of the National Academy of Sciences of the United States of America* **106**, 15732-15737, doi:10.1073/pnas.0906947106 (2009).

30 Dews, M. *et al.* The myc-miR-17~92 axis blunts TGF{beta} signaling and production of multiple TGF{beta}-dependent antiangiogenic factors. *Cancer research* **70**, 8233-8246, doi:10.1158/0008-5472.can-10-2412 (2010).

31 Bjork, J. K., Sandqvist, A., Elsing, A. N., Kotaja, N. & Sistonen, L. miR-18, a member of Oncomir-1, targets heat shock transcription factor 2 in spermatogenesis. *Development (Cambridge, England)* **137**, 3177-3184, doi:10.1242/dev.050955 (2010).

32 Shen, C. & Houghton, P. J. The mTOR pathway negatively controls ATM by up-regulating miRNAs. *Proceedings of the National Academy of Sciences of the United States of America* **110**, 11869-11874, doi:10.1073/pnas.1220898110 (2013).

33 Li, L., Shi, J. Y., Zhu, G. Q. & Shi, B. MiR-17-92 cluster regulates cell proliferation and collagen synthesis by targeting TGFB pathway in mouse palatal mesenchymal cells. *Journal of cellular biochemistry* **113**, 1235-1244, doi:10.1002/jcb.23457 (2012).

34 Liu, X. S. *et al.* MicroRNA-17-92 cluster mediates the proliferation and survival of neural progenitor cells after stroke. *The Journal of biological chemistry* **288**, 12478-12488, doi:10.1074/jbc.M112.449025 (2013).

35 Murakami, Y. *et al.* Comprehensive analysis of microRNA expression patterns in hepatocellular carcinoma and non-tumorous tissues. *Oncogene* **25**, 2537-2545, doi:10.1038/sj.onc.1209283 (2006).

36 Vreugdenhil, E. *et al.* MicroRNA 18 and 124a down-regulate the glucocorticoid receptor: implications for glucocorticoid responsiveness in the brain. *Endocrinology* **150**, 2220-2228, doi:10.1210/en.2008-1335 (2009).

37 Humphreys, K. J., Cobiac, L., Le Leu, R. K., Van der Hoek, M. B. & Michael, M. Z. Histone deacetylase inhibition in colorectal cancer cells reveals competing roles for members of the oncogenic miR-17-92 cluster. *Molecular carcinogenesis* **52**, 459-474, doi:10.1002/mc.21879 (2013).

38 Fox, J. L., Dews, M., Minn, A. J. & Thomas-Tikhonenko, A. Targeting of TGFbeta signature and its essential component CTGF by miR-18 correlates with improved survival in glioblastoma. *RNA (New York, N.Y.)* **19**, 177-190, doi:10.1261/rna.036467.112 (2013).

39 Chen, J. *et al.* MicroRNA-193b represses cell proliferation and regulates cyclin D1 in melanoma. *The American journal of pathology* **176**, 2520-2529, doi:10.2353/ajpath.2010.091061 (2010).

40 Li, X. F., Yan, P. J. & Shao, Z. M. Downregulation of miR-193b contributes to enhance urokinase-type plasminogen activator (uPA) expression and tumor progression and invasion in human breast cancer. *Oncogene* **28**, 3937-3948, doi:10.1038/onc.2009.245 (2009).

41 Mao, K. *et al.* Restoration of miR-193b sensitizes Hepatitis B virus-associated hepatocellular carcinoma to sorafenib. *Cancer letters* **352**, 245-252, doi:10.1016/j.canlet.2014.07.004 (2014).

42 Xu, C. *et al.* MicroRNA-193b regulates proliferation, migration and invasion in human hepatocellular carcinoma cells. *European journal of cancer (Oxford, England : 1990)* **46**, 2828-2836, doi:10.1016/j.ejca.2010.06.127 (2010).

43 Skalsky, R. L. *et al.* The viral and cellular microRNA targetome in lymphoblastoid cell lines. *PLoS pathogens* **8**, e1002484, doi:10.1371/journal.ppat.1002484 (2012).

44 Ji, J. *et al.* Let-7g targets collagen type I alpha2 and inhibits cell migration in hepatocellular carcinoma. *Journal of hepatology* **52**, 690-697, doi:10.1016/j.jhep.2009.12.025 (2010).

45 Lan, F. F. *et al.* Hsa-let-7g inhibits proliferation of hepatocellular carcinoma cells by downregulation of c-Myc and upregulation of p16(INK4A). *International journal of cancer* **128**, 319-331, doi:10.1002/ijc.25336 (2011).

46 Shell, S. *et al.* Let-7 expression defines two differentiation stages of cancer. *Proceedings of the National Academy of Sciences of the United States of America* **104**, 11400-11405, doi:10.1073/pnas.0704372104 (2007).

47 Boyerinas, B. *et al.* Identification of let-7-regulated oncofetal genes. *Cancer research* **68**, 2587-2591, doi:10.1158/0008-5472.can-08-0264 (2008).

48 Shimizu, S. *et al.* The let-7 family of microRNAs inhibits Bcl-xL expression and potentiates sorafenib-induced apoptosis in human hepatocellular carcinoma. *Journal of hepatology* **52**, 698-704, doi:10.1016/j.jhep.2009.12.024 (2010).

49 Chen, Z. *et al.* Hypoxia-responsive miRNAs target argonaute 1 to promote angiogenesis. *The Journal of clinical investigation* **123**, 1057-1067, doi:10.1172/jci65344 (2013).

50 Qian, P. *et al.* Pivotal role of reduced let-7g expression in breast cancer invasion and metastasis. *Cancer research* **71**, 6463-6474, doi:10.1158/0008-5472.can-11-1322 (2011).

51 Romania, P. *et al.* MicroRNA 155 modulates megakaryopoiesis at progenitor and precursor level by targeting Ets-1 and Meis1 transcription factors. *British journal of haematology* **143**, 570-580, doi:10.1111/j.1365-2141.2008.07382.x (2008).

52 Ceppi, M. *et al.* MicroRNA-155 modulates the interleukin-1 signaling pathway in activated human monocyte-derived dendritic cells. *Proceedings of the National Academy of Sciences of the United States of America* **106**, 2735-2740, doi:10.1073/pnas.0811073106 (2009).

53 Chen, Y. *et al.* 1,25-Dihydroxyvitamin D promotes negative feedback regulation of TLR signaling via targeting microRNA-155-SOCS1 in macrophages. *Journal of immunology (Baltimore, Md. : 1950)* **190**, 3687-3695, doi:10.4049/jimmunol.1203273 (2013).

54 Pedersen, I. M. *et al.* Onco-miR-155 targets SHIP1 to promote TNFalpha-dependent growth of B cell lymphomas. *EMBO molecular medicine* **1**, 288-295, doi:10.1002/emmm.200900028 (2009).

55 Rai, D., Kim, S. W., McKeller, M. R., Dahia, P. L. & Aguiar, R. C. Targeting of SMAD5 links microRNA-155 to the TGF-beta pathway and lymphomagenesis. *Proceedings of the National Academy of Sciences of the United States of America* **107**, 3111-3116, doi:10.1073/pnas.0910667107 (2010).

56 Yin, Q. *et al.* MicroRNA miR-155 inhibits bone morphogenetic protein (BMP) signaling and BMP-mediated Epstein-Barr virus reactivation. *Journal of virology* **84**, 6318-6327, doi:10.1128/jvi.00635-10 (2010).

57 Neilsen, P. M. *et al.* Mutant p53 drives invasion in breast tumors through up-regulation of miR-155. *Oncogene* **32**, 2992-3000, doi:10.1038/onc.2012.305 (2013).

58 Liu, W. J. *et al.* MLH1 as a direct target of MiR-155 and a potential predictor of favorable prognosis in pancreatic cancer. *Journal of gastrointestinal surgery : official journal of the Society for Surgery of the Alimentary Tract* **17**, 1399-1405, doi:10.1007/s11605-013-2230-5 (2013).

59 Du, Z. M. *et al.* Upregulation of MiR-155 in nasopharyngeal carcinoma is partly driven by LMP1 and LMP2A and downregulates a negative prognostic marker JMJD1A. *PLoS One* **6**, e19137, doi:10.1371/journal.pone.0019137 (2011).

60 Valeri, N. *et al.* Modulation of mismatch repair and genomic stability by miR-155. *Proceedings of the National Academy of Sciences of the United States of America* **107**, 6982-6987, doi:10.1073/pnas.1002472107 (2010).

61 Schotte, D. *et al.* Discovery of new microRNAs by small RNAome deep sequencing in childhood acute lymphoblastic leukemia. *Leukemia* **25**, 1389-1399, doi:10.1038/leu.2011.105 (2011).

62 Godfrey, A. C. *et al.* Serum microRNA expression as an early marker for breast cancer risk in prospectively collected samples from the Sister Study cohort. *Breast Cancer Res* **15**, R42, doi:10.1186/bcr3428 (2013).

63 Ottman, R., Nguyen, C., Lorch, R. & Chakrabarti, R. MicroRNA expressions associated with progression of prostate cancer cells to antiandrogen therapy resistance. *Mol Cancer* **13**, 1, doi:10.1186/1476-4598-13-1 (2014).
